# Multimodal Regulation of NET Formation in Pregnancy: Progesterone Antagonizes the Pro-NETotic Effect of Estrogen and G-CSF

**DOI:** 10.3389/fimmu.2016.00565

**Published:** 2016-12-05

**Authors:** Stavros Giaglis, Maria Stoikou, Chanchal Sur Chowdhury, Guenther Schaefer, Franco Grimolizzi, Simona W. Rossi, Irene Mathilde Hoesli, Olav Lapaire, Paul Hasler, Sinuhe Hahn

**Affiliations:** ^1^Department of Biomedicine, University Hospital Basel, Basel, Switzerland; ^2^Department of Rheumatology, Cantonal Hospital Aarau, Aarau, Switzerland; ^3^Department Clinical Sciences, Polytechnic University Marche, Ancona, Italy; ^4^University Women’s Hospital, University Hospital Basel, Basel, Switzerland

**Keywords:** neutrophils, NETs, pregnancy, sex hormones, G-CSF

## Abstract

Human pregnancy is associated with a mild pro-inflammatory state, characterized by circulatory neutrophil activation. In order to explore the mechanism underlying this alteration, we examined NETosis during normal gestation. Our data indicate that neutrophils exhibit a pro-NETotic state, modulated in a multimodal manner during pregnancy. In general, circulatory granulocyte colony-stimulating factor, the levels of which increase during gestation, promotes neutrophil extracellular trap (NET) formation. Early in pregnancy, NETosis is enhanced by chorionic gonadotropin, whereas toward term is stimulated by estrogen. A complex interaction between estrogen and progesterone arises, wherein progesterone restrains the NETotic process. In this state, extensive histone citrullination is evident, yet full NETosis is inhibited. This coincides with the inability of neutrophil elastase to translocate from the cytoplasm to the nucleus and is regulated by progesterone. Our findings provide new insight concerning gestational and hormone-driven pathologies, since neutrophil recruitment, activation, and NET release could be associated with excessive endothelial and placental injury.

## Introduction

Pregnancy presents a unique challenge for the maternal immune system, which is modulated in such a manner that the mother accepts her semi-allogeneic fetus, yet is still capable of mounting an effective response against infections (1, 2). Although a considerable body of knowledge has been accumulated regarding the role of T lymphocytes, regulatory T cells, or natural killer cells in tolerance promotion or tissue modification during gestation, the role of the innate immune response and the involvement of neutrophils in particular has not been examined to the same extent in normal pregnancy (3). There is a need to address this, since a comprehensive body of evidence implicates aberrant neutrophil activity in severe pregnancy complications such as preeclampsia (PE), recurrent fetal loss, or poor pregnancy outcome due to autoimmune conditions such as systemic lupus erythematosus (SLE) (4–8). In part, these pathologies appear to entail an excessive alteration of the moderately increased inflammatory activity of circulatory neutrophils in normal pregnancy (4, 9). Such overt neutrophil activity is proposed to contribute to increased neutrophil extracellular traps (NETs) in preeclamptic placentae or, as recently shown, those affected by SLE (10–12).

Neutrophils play a key role in the immediate response to infections, employing an array of weapons including phagocytosis, release of toxic granular enzymes and of reactive oxygen species (ROS), or the generation of NETs to dispose of harmful microorganisms (13, 14). NETs are a rather unique innate immunity tool, being formed by the extrusion of nuclear DNA into the extracellular environment, where they ensnare a wide array of microorganisms, ranging from bacteria and fungi to parasites (15). Aberrant NET formation may induce damage or cell death of neighboring tissues and has been implicated in a number of pathologies including rheumatoid arthritis (RA), SLE, small vessel vasculitis, or coagulopathies (16). The underlying signal-transducing pathway initiating NETosis involves calcium mobilization, generation of ROS by NAPDH oxidase, nuclear transfer of neutrophil elastase (NE), myeloperoxidase (MPO), and peptidylarginine deiminase 4 (PAD4), and histone citrullination by the latter (17–21). These events contribute to chromatin unfolding, a prerequisite for efficient DNA extrusion (21–23).

To date, the generation of NETs and the regulation of their release have not been studied during the three trimesters of normal human pregnancy (7, 12). Our data indicate that neutrophils from normal healthy pregnancies exhibit a distinctive pro-NETotic phenotype, which increases toward term. It was determined that granulocyte colony-stimulating factor (G-CSF) not only contributes to increasing neutrophil counts during gestation but also to progressively enhanced NETosis. Early in gestation, NETosis is augmented by the action of human chorionic gonadotropin (hCG), while toward term the steroid sex hormones estradiol (E2) and progesterone (P4) modulated neutrophil activity in a complex manner. While E2 acts to promote NET formation, P4 acts as an antagonist, by retaining neutrophils in an advanced primed state, thus hindering progression of NETosis. Our findings suggest that the regulatory mechanism evoked by P4 involves the prevention of NE transfer from the cytoplasm to the nucleus, a step previously demonstrated to be vital for effective NET formation (18).

## Materials and Methods

### Human Subjects

Pregnant women were recruited at the time of their routine examination at the end of the first (median gestational age: 12 weeks and 4 days – *n* = 15; median age: 34.1 years) and second trimesters (median gestational age: 24 weeks and 3 days – *n* = 25; median age: 34.1 years) and at the time of elective cesarean section toward the end of the third trimester (median gestational age at delivery: 38 weeks and 4 days – *n* = 35; median age: 34.1 years) (Table S1 in Supplementary Material). Healthy non-pregnant controls, matched for age (*n* = 45; median age: 33.5 years), were recruited at the Blood Bank of the Swiss Red Cross, Basel (Table S1 in Supplementary Material). Inclusion criteria for non-pregnant controls were fair general condition, female sex, age ≥25 and ≤45 years, and for blood donors fulfilling national criteria for blood donation. Exclusion criteria were current or previous systemic autoimmune disease, asthma, reconvalescence after major illness, surgery, current medication with corticosteroids, immunosuppressive agents and malignant neoplasia, or chemotherapy within 5 years before recruitment for the study. Exclusion criteria for pregnant subjects included any major complication of pregnancy or coincident disease, such as PE, pre- or post-term labor (<37 or >42 weeks), intrauterine growth retardation, and viral, bacterial, or parasitic infections. Informed, written consent was obtained from all subjects prior to inclusion in the study, which was approved by the Ethical Review Board of Basel/Basel-Land, Switzerland.

### Blood Cell Count and Preparation of Plasma and Serum

Whole blood was collected into EDTA- and silicone-coated tubes (Sarstedt), and 25 μl of blood was analyzed by a Hemavet 950FS (Drew Scientific) for complete blood cell counts. Plasma and serum was collected and processed as described previously (24). Samples were studied immediately or stored at −80°C until analyzed.

### Human Neutrophil Isolation

Neutrophils were isolated by Dextran–Ficoll density centrifugation (25). Cell viability was assessed by trypan blue dye exclusion via a hemocytometer and was measured routinely 96–98% with a purity of over 95%. Neutrophils were directly seeded in 24-well or 96-well plates and allowed to settle for 15 min at 37°C under 5% CO_2_ prior to further experimentation. Time-points of measurements are given in the figure legends.

### Stimulation and Neutralization Studies

For *in vitro* incubation studies, 2.5 × 10^4^ neutrophils from healthy women were treated with 3% serum or 6% plasma derived from non-pregnant controls and pregnant donors during the first, second, and third trimesters of gestation. All experiments were carried out over 3 h in four to six replicates.

Neutrophils from healthy controls were incubated with ascending concentrations of hCG, E2, E3, P4, or G-CSF, which covered the physiological plasma concentrations during gestation, individually or in different combinations.

For the two-step stimulation *in vitro* experiments, neutrophils were pretreated with hormones or G-CSF as a primary stimulus for 60 min, and then exposed to the secondary stimulus (PMA or G-CSF) for another 120 min, for a total time of 3 h.

To neutralize sex hormones’ activity, pooled sera or plasma from the study groups of interest were pretreated for 30 min with fulvestrant (10 μg/ml, Sigma) and mifepristone (10 μg/ml, Sigma) and used for a 3-h treatment of control neutrophils for the inhibition of estrogen and progestin receptors, respectively. To neutralize G-CSF, pooled sera or plasma from the study groups of interest were pretreated with anti-G-CSF antibody (0.2 μg/ml, Peprotech) for 30 min.

### Fluorimetric Quantification and Fluorescence Microscopy

NETs were quantified by SytoxGreen fluorimetry (10, 20, 26). 2.5 × 10^4^ freshly isolated neutrophils were cultured in the presence of 0.2 μM SytoxGreen (Invitrogen, Life Technologies) in a 96-well dark microtiter plate at 37°C under 5% CO_2_ and left untreated or stimulated with the aforementioned agents over 3 h. PMA (25nM) was used as the positive control. Fluorescence (excitation 485 nm, emission 535 nm) was measured in a Biotek Synergy H1 Hybrid Reader (Biotek) and results given as DNA mean fluorescence intensity (MFI). Photomicrographs in bright field and green fluorescence spectra were assessed with an Olympus IX50 inverted fluorescence microscope coupled to an Olympus XM10 monochromatic CCD camera and analyzed with the Olympus CellSens Dimension software (Olympus).

### Neutrophil Viability

Apoptosis was detected by Annexin V/7-aminoactinomycin D (7-AAD) staining (BD BioSciences) according to the manufacturer’s instructions. 10^4^ cells were counted by flow cytometry using a BD Accuri™ C6 flow cytometer (Becton-Dickinson). The data were analyzed using Flowjo v10 software (Treestar).

### Cytokine Proteome Array

Cytokines, chemokines, and acute phase proteins were detected with the Human Cytokine Array Kit (R&D Systems) according to the manufacturer’s instructions. Pooled sera collected from control non-pregnant individuals and pregnant donors during the first, second, and third trimesters of gestation were centrifuged and incubated with the pre-coated nitrocellulose membranes. After washing and addition of the detection antibody streptavidin–HRP conjugates, the membranes were exposed to X-ray film (Fuji). The cytokine proteomic array comprised 36 targets spotted in duplicate on the membranes. The intensity of each spot in the captured images was analyzed with ImageJ analysis software (NIH Image Processing).

### NE, MPO, Cell-Free Histone/DNA Complex, MPO/DNA Complex, and G-CSF Protein Analysis

The concentrations of NE and MPO were measured in sera and plasma by sandwich ELISA, utilizing, respectively, the Elastase/a1-PI Complex ELISA Kit (Calbiochem) and the human MPO ELISA Kit. Histone/DNA complexes in sera and plasma were measured using the Human Cell Death Detection ELISA^PLUS^ (Roche Diagnostics); nucleosomes in cell culture supernatants were detected similarly after incubation with DNase I (10 U for 5 min) (Roche Diagnostics). To identify NET-associated MPO/DNA complexes, a modified capture ELISA was utilized (27). NET-associated MPO in culture supernatant was captured using the coated 96-well plate of the human MPO ELISA Kit (Hycult Biotech), and the NET-associated DNA backbone was detected using the anti-DNA-POD antibody of the Human Cell Death Detection ELISA^PLUS^ (Roche Diagnostics). G-CSF serum and plasma protein concentrations were assessed with the Human G-CSF Quantikine ELISA Kit (R&D Systems).

### Oxidative Burst Analysis

Nicotinamide adenine dinucleotide phosphate (NADPH) oxidase-mediated ROS production was measured either by a 2′,7′-dichloro dihydrofluorescein diacetate (DCFH-DA) plate assay (28) or a luminol-based chemiluminescence microtiter plate assay (29, 30). Also, 2.5 × 10^4^ neutrophils per well were incubated without or with stimulants mentioned above in dark 96-well microtiter plates with 25 μM DCFH-DA (Sigma-Aldrich), which reacts with ROS species produced in intracellular compartments (granules or phagosomes). Fluorescence was recorded immediately in a Biotek Synergy H1 Hybrid plate Reader (Biotek) for 30 min. The response was expressed as relative fluorescence units (RFU). Similarly, 2.5 × 10^4^ neutrophils per well were incubated without or with the aforementioned stimulants in white 96-well microtiter plates with 60μM luminol (5-amino-2,3-dihydro 1,4-phthalazinedione). Chemiluminescence was recorded every 5 min over a period of 30 min in a Biotek Synergy H1 Hybrid plate Reader (Biotek), and the response was expressed as relative luminescence units (RLU).

### Immunohistochemistry, Morphometric Analysis, and Confocal Microscopy

The 1 × 10^5^ neutrophils were seeded on poly-l-lysine-coated glass coverslips (BD Biosciences) in 24-well tissue-culture plates and allowed to settle prior to stimulation as described above. Coverslips were rinsed with ice-cold HBSS and the cells fixed with 4% paraformaldehyde and blocked overnight (HBSS with 10% FBS, 0.1% Tween20, and 2mM EDTA) at 4°C. NETs were detected with rabbit anti-NE (Abcam), rabbit anti-MPO (Dako), and rabbit anti-citrullinated histone H3 (citH3, Abcam). Secondary antibodies were goat anti-rabbit IgG AF555, goat anti-rabbit IgG AF488 (Invitrogen Life Technologies), and goat anti-mouse IgG AF647. DNA was stained with 4′,6-diamidino-2-phenylindole (DAPI, Sigma-Aldrich). NETs were visualized by using a Zeiss Axioplan 2 Imaging fluorescence microscope in conjunction with a Zeiss AxioCam MRm monochromatic CCD camera and analyzed with Axiovision 4.8.2 software (Carl Zeiss). A minimum of 20 fields (at least 1,000 neutrophils) per case was evaluated for MPO/NE and DNA co-staining; nuclear phenotypes and NETs were counted and expressed as percentage of the total number of cells in the fields.

In another setup, NETs were quantified by IHC staining of 2.5 × 10^4^ neutrophils per well in a 96-well plate with mouse anti-human MPO antibody (Abcam) and rabbit anti-human citH3 antibody (Abcam), or the respective isotype controls, followed by incubation with goat anti-mouse IgG AF555 and goat anti-rabbit IgG AF488 (Invitrogen Life Technologies). DNA was counterstained with DAPI (Sigma-Aldrich). NETs were visualized by using an Olympus IX81 motorized epifluorescence microscope (Olympus) in conjunction with an Olympus XM10 monochromatic CCD camera (Olympus) and analyzed with the Olympus CellSens Dimension software (Olympus). A minimum of 20 fields at 10× magnification (at least 500–1,000 neutrophils) per sample were evaluated for MPO/citH3 and DNA co-staining through ImageJ analysis software (NIH); nuclear phenotypes and NETs were determined, counted, and expressed as percentage of the total area of cells in the fields (31). Images were captured on a Nikon A1R inverted microscope (Nikon) coupled to a Visitron CSU-W1 spinning disk confocal microscopy module (Visitron) and a Thor ablation laser (Thor Labs) using an UPL APO 60×/1.40 oil objective lens with the Visiview Cell Analyser software (Visitron Systems, Version 3.1.2.2).

### Phagocytosis Activity

Neutrophil phagocytic activity was examined by the uptake of latex beads coated with FITC-labeled rabbit IgG into cells (Cayman Chemical) according to the instruction manual. The 1 × 10^5^ untreated neutrophils exposed to various stimulants were resuspended in 200 μl phagocytosis buffer to which FITC-labeled beads (1:100) were added and incubated for 2 h at 37°C. The amount of phagocytosis was determined by flow cytometry utilizing the BD Accuri™ C6 flow cytometer (Becton-Dickinson) and analyzed by Flowjo v10 software (Treestar). The uptake of the beads into neutrophils was additionally captured with an Olympus IX50 inverted fluorescence microscope and phagocytosis quantified as described above with ImageJ analysis software.

### RNA Isolation and Quantitative Real-time PCR

Total RNA was isolated from 3 × 10^6^ neutrophils by using the RNeasy Mini Kit (Qiagen). TaqMan real-time quantitative RT-PCR was performed utilizing the Applied Biosystems StepOne Plus cycler (Applied Biosystems) and TaqMan Gene Expression Assay primer and probe sets (Applied Biosystems) for *ELANE* (HS00236952_m1), *MPO* (HS00924296_m1), *PAD4* (HS00202612_m1), IL-6 (HS00202612_m1), IL-8 (HS00202612_m1), and *TNF (*HS00202612_m1). Data were normalized to the housekeeping gene *B2M* (HS99999907_m1), after a selection procedure from six different endogenous reference genes, as suggested in the MIQE guidelines (32). Relative values were calculated with 2^−DDCt^ analysis (33).

### Protein Isolation and Western Blot Analysis for PAD4 and Citrullinated Histone H3

Total protein was isolated by NucleoSpin TriPrep kit (Macherey-Nagel) from 5 × 10^6^ neutrophils. All protein concentrations were determined with the MN Protein Quantification Assay (Macherey-Nagel). Western blotting was performed utilizing AnykD Mini-PROTEAN TGX Gels (Biorad) and nylon/nitrocellulose membranes (Biorad). Primary and secondary antibodies utilized were rabbit anti-MPO (Cell Signalling Technologies), rabbit anti-PAD4 (Abcam), rabbit anti-citH3 (Abcam), mouse anti-β-Actin (Sigma), anti-rabbit HRP (Santa Cruz), and anti-mouse HRP (Santa Cruz). HRP activity was detected by using SuperSignal West Pico Chemiluminescent Substrate (Thermo Scientific). Equal loading was verified using beta-actin. Western blots of citrullinated H3 (citH3) protein were prepared as described previously (34). Gel documentation, densitometric analysis, and protein quantification of the western blots was performed using the ChemiDoc XRS+ imaging system (Biorad) with the ImageLab 4.1 image analysis software (Biorad).

### Statistical Analysis

All data are presented as mean ± SE. Descriptive statistics for continuous parameters consisted of median and range, and categorical variables were expressed as percentages. Comparisons between patients and healthy controls were carried out by the Mann–Whitney *U* test with a Welch post-test correction. Statistical significance in multiple comparisons was by one-way analysis of variance (ANOVA) with a Dunn’s post-test correction. *P* values under <0.05 were considered statistically significant. Data were processed in GraphPad Prism version 6.0 for MacOSX (GraphPad Software Inc.^1^). Professional statistical assistance was provided by Andreas Schoetzau.^2^

## Results

### Pregnancy Is Associated with a Pro-NETotic State That Increases with Advancing Gestation

**Figure 1:**
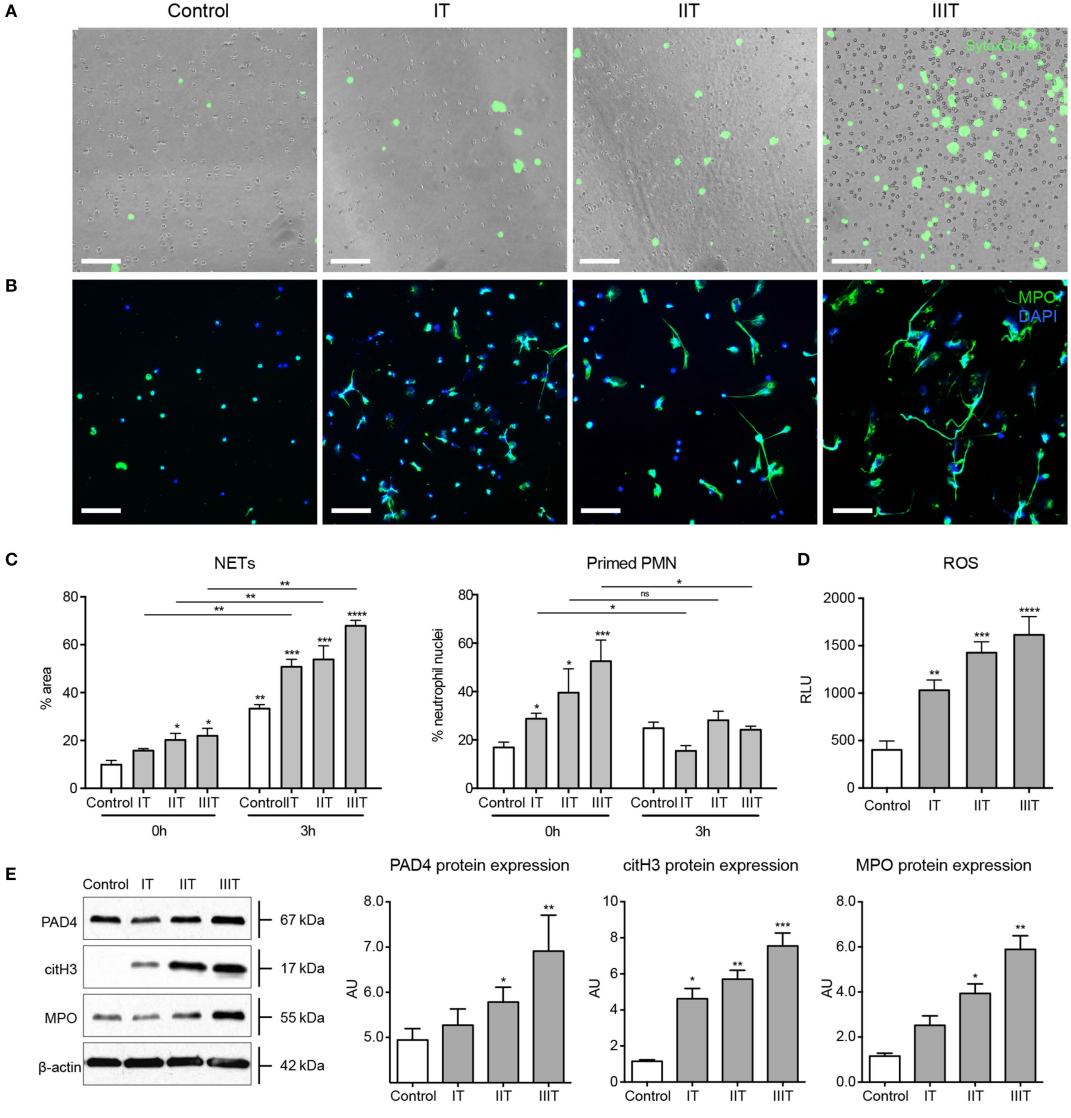

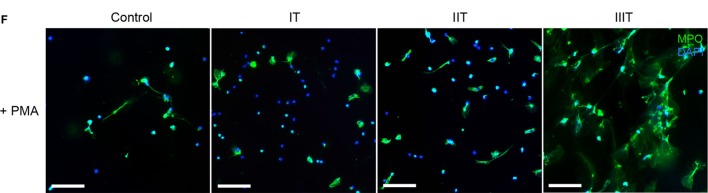
**NET formation and neutrophil pro-NETotic priming are augmented during pregnancy**. **(A)** *In vitro* spontaneous NET formation by neutrophils from healthy pregnant donors during the three consecutive trimesters of their pregnancies over a 3-h time course detected by fluorescence microscopy utilizing the dsDNA-binding fluorescent SytoxGreen dye. Scale bars: 100 μm. **(B)** Immunofluorescence staining of neutrophils from healthy pregnant donors during the three trimesters of gestation for MPO (green) and DNA counterstain with DAPI (blue) after 3 h of culture (3 h) compared to non-pregnant healthy donors. Scale bars: 50 μm. **(C)** Morphometric analysis of the NETotic and pro-NETotic primed neutrophils from healthy female controls and pregnant donors during the three trimesters of gestation at the baseline steady state (0 h) and over a 3-h time course (3 h). **(D)** Oxidative burst in neutrophils from healthy control donors and donors during pregnancy during the three consecutive trimesters over a 3-h time course as monitored by DCFH-DA detection. **(E)** Western blot and densitometric analysis of PAD4, citH3, MPO, and beta-actin protein expression levels in neutrophil lysates from healthy female controls and donors during the three trimesters of their pregnancies. **(F)** Fluorescent immunocytochemistry for MPO (green) and DNA counterstain with DAPI (blue) of neutrophils from healthy non-pregnant female controls and pregnant donors during the three trimesters of gestation after incubation for 2 h with 25nM of the NET-inducing agent phorbol-12-myristate-13-acetate (PMA). Scale bars: 50 μm. Data are presented as mean ± SEM. *P < 0.05, **P < 0.01, ***P < 0.001, and ****P < 0.0001 (one or two way ANOVA followed by Bonferroni’s multiple comparison post-test). All experiments were performed at least six times with consistent results. IT, first trimester; IIT, second trimester; IIIT, third trimester; P, pregnancy; RFU, relative fluorescence units; AU, arbitrary units.

An initial examination of circulatory neutrophils isolated from normal maternal blood samples with SytoxGreen, a cell impermeable DNA dye, indicated that they exhibited an increased tendency to form NETs *in vitro*, when compared to matching non-pregnant control donors (Figure 1A; Video S1 in Supplementary Material). This was most evident after culturing for 3 h, as confirmed by immunocytochemical staining (Figure 1B). To verify that these filamentous structures stained by SytoxGreen were indeed NETs, we used immunofluorescence microscopy (Figure 1B) and detection of MPO/cell-free DNA complexes in culture supernatants (Figure S1A in Supplementary Material). This confirmed the increased tendency for neutrophils to undergo increased NETosis with advancing gestational age. While the numbers of neutrophils increased during pregnancy, the proportion undergoing NETosis was greater than the increment in neutrophil numbers (Figure S1B in Supplementary Material).

Analysis of nuclear shape indicated that a significant proportion of the circulatory neutrophils in pregnant women were in a primed pro-NETotic state, evident by a delobulated diffused staining pattern (Figure 1C). This enhanced pro-NETotic activity was mirrored by an increase in ROS production (Figure 1D), as well as by MPO expression and histone H3 citrullination, likely driven by enhanced PAD4 expression (Figure 1E), key signaling events required for efficient NET generation. This pro-NETotic phenotype was further substantiated by monitoring the response to other stimuli, such as that to phorbol ester (PMA), where a hyperresponsive increase in NET generation was observed, particularly evident in samples collected close to term (Figure 1F; Figures S1C,D in Supplementary Material).

### G-CSF Promotes a Pro-NETotic Status during Pregnancy

**Figure 2:**
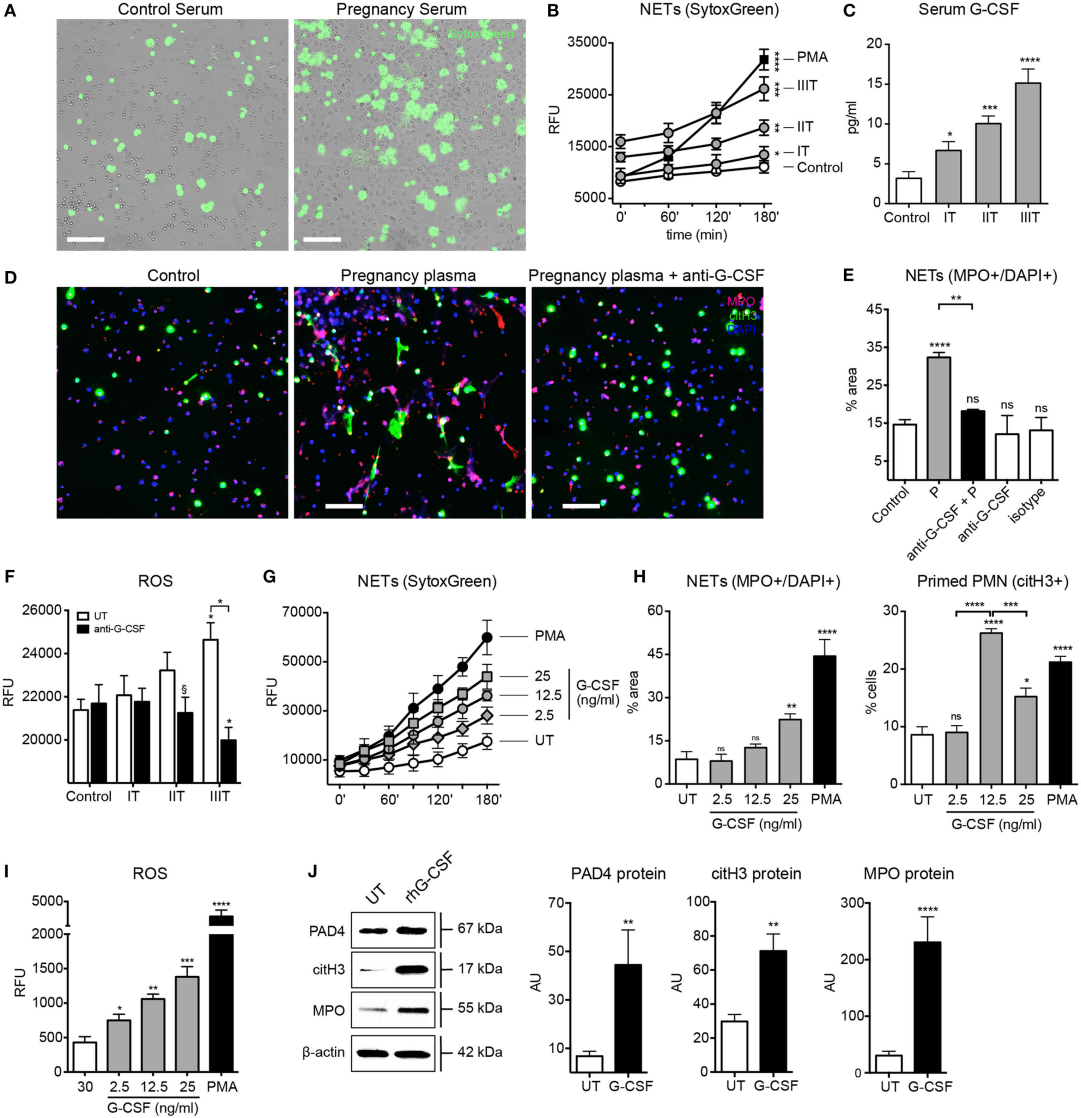
**G-CSF leads to neutrophil pro-NETotic priming during the course of pregnancy**. **(A)** *In vitro* spontaneous extracellular DNA release monitored over a 3-h time course by SytoxGreen dye fluorescence microscopy after treatment of control neutrophils with pooled sera from women during their pregnancy. Scale bars: 100 μm. **(B)** Fluorimetric quantification of extracellular DNA release from neutrophils treated *in vitro* with serum from the three trimesters of pregnancy using dsDNA-binding SytoxGreen dye compared to the NET-inducing phorbol ester PMA (25nM). **(C)** Detection of G-CSF levels in sera from donors during the three trimesters of pregnancy by EIA. **(D)** Fluorescent immunostaining for MPO (red), citH3 (green), and DNA (blue) depicting the formation of NETs after *in vitro* treatment of control neutrophils with 6% pooled plasma from women during their pregnancy and 30 min pretreatment of pregnancy plasma with 50 ng/ml anti-G-CSF neutralizing antibody compared to plasma from control non-pregnant individuals. Scale bars: 50 μm. **(E)** Quantitative morphometric analysis of the NETotic (MPO+/DNA+) positive and pro-NETotic primed (citH3+) neutrophils from healthy non-pregnant donors under the aforementioned experimental setup. **(F)** Intracellular ROS generation from neutrophils cocultured *in vitro* with 6% plasma derived from donors during the three trimesters of pregnancy and 50 ng/ml anti-G-CSF neutralizing antibody monitored with DCFH-DA. **(G)** Evaluation of rhG-CSF activity by *in vitro* titration experiments using fluorimetric analysis of control neutrophils after *in vitro* incubation with 2.5, 12.5, and 25 ng/ml rhG-CSF compared to untreated (UT) and treated with PMA (25nM) neutrophils. **(H)** Morphometric analysis of the NETotic (MPO+/DNA+) and pro-NETotic primed (citH3+) neutrophils from healthy control donors under rhG-CSF treatment *in vitro* at the indicated concentrations compared to untreated (UT) and PMA-treated neutrophils. **(I)** Oxidative burst in neutrophils from healthy control donors treated *in vitro* with rhG-CSF at the indicated concentrations monitored by DCFH-DA detection and compared to untreated (UT) and PMA-treated neutrophils. **(J)** Western blot and densitometric analysis of PAD4, citH3, MPO, and beta-actin protein expression in neutrophil lysates from healthy female controls treated *in vitro* with the physiological concentrations of rhG-CSF (12.5 ng/ml). Data are presented as mean ± SEM. *P < 0.05, **P < 0.01, ***P < 0.001, and ****P < 0.0001 (one or two way ANOVA followed by Bonferroni’s multiple comparison post-test). All experiments were performed at least six times with consistent results. IT, first trimester; IIT, second trimester; IIIT, third trimester; RFU, relative fluorescence units.

In order to assess which circulatory factors could contribute to the pro-NETotic state, we examined the effect of sera from pregnant women on control neutrophils, where we observed that such treatment augmented NET formation (Figure 2A), and that in a gestational age-dependent manner (Figure 2B). G-CSF was determined to be significantly elevated in a cytokine proteome array analysis (Figure S2 in Supplementary Material), a feature confirmed by specific enzyme immunoassay (EIA) (Figure 2C). This piqued our interest, as this cytokine has recently been implicated to predisposing neutrophils toward NETosis in a murine tumor model (35). Circulatory G-CSF appears to be an important contributor to the pro-NETotic state present in pregnancy, as a G-CSF blocking antibody diminished NET formation induced by plasma from pregnant women (Figures 2D,E). This was greatest with advanced gestation, indicating increased quantities of circulatory G-CSF close to term (Figure 2C). On the other hand, treatment of maternal plasma samples with the anti-G-CSF antibody led to a step-wise decrease in ROS production inversely proportional to advancing gestational age (Figure 2F). The pro-NETotic role of circulatory G-CSF during pregnancy was investigated by assessing the effect of different concentrations of recombinant human (rh)G-CSF on isolated normal neutrophils. rhG-CSF increased NET formation in a concentration-dependent manner (Figure 2G; Figure 2H, left panel), while 12.5 mg/ml, which corresponds to the physiologic range in pregnancy, triggered the highest primed pro-NETotic state (Figure 2H, right panel) that was highly responsive to a secondary stimulus by PMA (data not shown). Furthermore, increasing doses of rhG-CSF led to concordant increases in ROS production (Figure 2I), while physiological concentrations led to increases in key NETosis signaling components, namely PAD4, citH3, and MPO (Figure 2J). These data suggest that G-CSF plays a vital role in promoting a pro-NETotic phenotype in pregnancy.

### The Gestational Hormonal Milieu Regulates NET Formation

**Figure 3:**
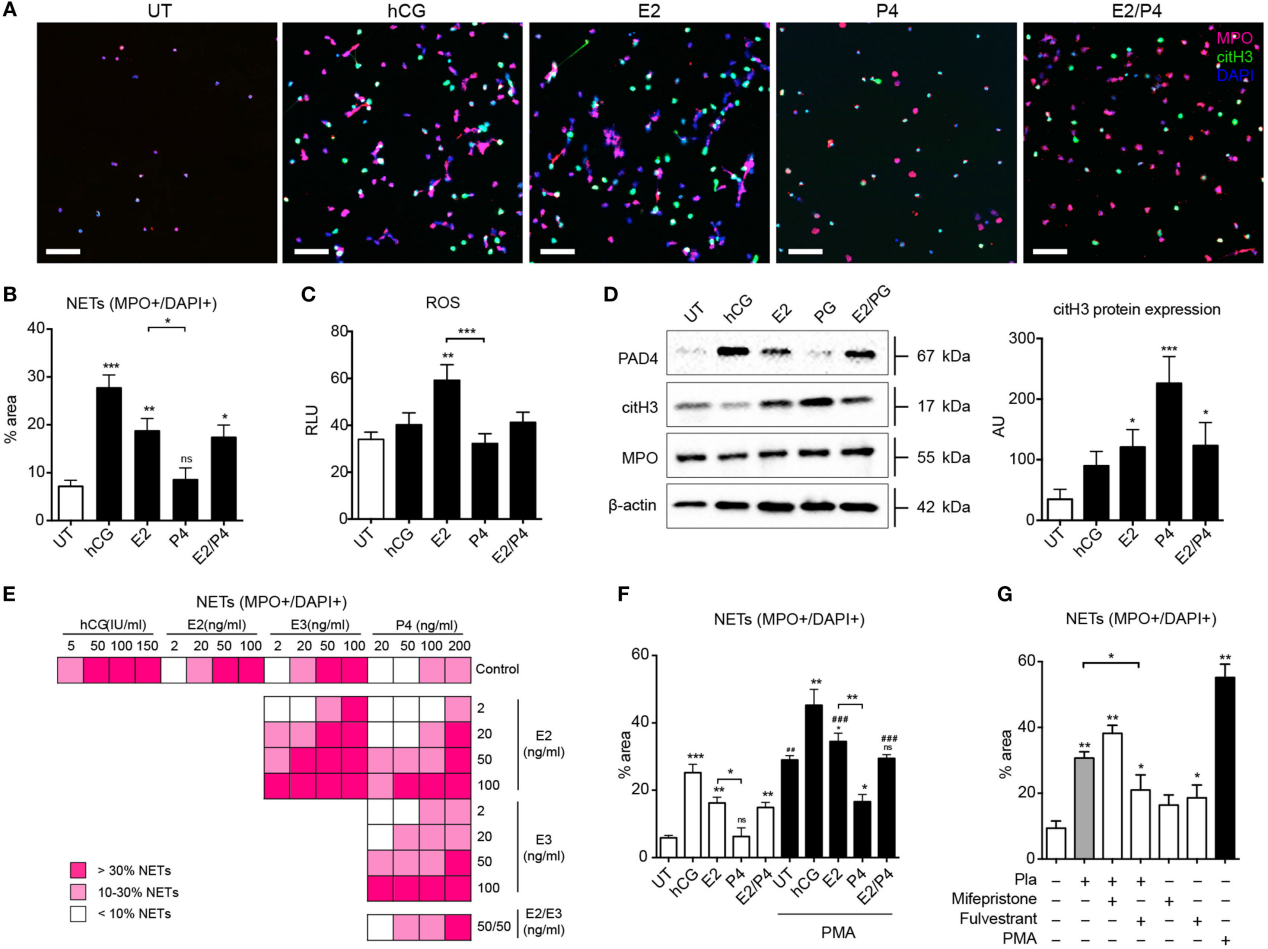
**Neutrophil *in vitro* pro-NETotic priming is regulated by pregnancy hormones**. **(A)** Fluorescent immunocytochemistry of peripheral neutrophils after incubation with physiologic pregnancy concentrations of the sex hormones hCG (50 IU/ml), E2 (20 ng/ml), P4 (50 ng/ml), and the combination of 20 ng/ml E2 and 50 ng/ml P4 for MPO (red), citH3 (green), and DNA counterstain with DAPI (blue) after a 2-h *in vitro* culture. **(B)** Morphometric analysis of the NETotic (MPO+/DAPI+) neutrophils from healthy control donors under the aforementioned hormonal *in vitro* treatment. **(C)** Oxidative burst in neutrophils from healthy control donors treated with physiologic pregnancy concentrations of hCG, E2, P4, and the combination of E2 and P4 for 3 h by DCFH-DA. **(D)** Western blot analysis of PAD4, citH3, MPO, and beta-actin protein expression levels in lysates from healthy female control neutrophils treated *in vitro* with the aforementioned concentrations of sex hormones. **(E)** Information graphic summarizing the *in vitro* spontaneous NET release monitored by dsDNA-binding fluorescent SytoxGreen dye in a 3-h time course after treatment with four ascending concentrations of each of the gestational hormones hCG, E2, E3, and P4, separately or in crosswise combinations. **(F)** Morphometric analysis of the NETotic (MPO+/DAPI+) and pro-NETotic primed (citH3+) neutrophils from healthy non-pregnant donors after 1 h pretreatment with physiologic concentrations of hCG, E2, P4, and E2/P4 and stimulation with 25nM of the NET-inducing agent PMA as a second hit for additional 2 h *in vitro*. **(G)** Detection of *in vitro* spontaneous NET formation of neutrophils from healthy controls cocultured with pregnancy plasma pretreated with 10 μg/ml of the specific inhibitors of the estrogen and progesterone receptors, fulvestrant and mifepristone (RU486), respectively, over a 3-h time course by fluorescence microscopy using morphometric analysis of the NETotic (MPO+/DAPI+) and pro-NETotic primed (citH3+) neutrophils. Data are presented as mean ± SEM. *P < 0.05, **P < 0.01, ***P < 0.001, and ****P < 0.0001 (one or two way ANOVA followed by Bonferroni’s multiple comparison post-test). All experiments were performed at least six times with consistent results. RFU, relative fluorescence units; Pla, pregnancy plasma.

The immune-modulatory role of hCG, estrogen, and progesterone during pregnancy is well documented. hCG levels peak at the end of the first trimester, while those of estrogen and progesterone reach a maximum at term (36–38). Accordingly, we examined their influence, focusing on the interplay between estrogen and progesterone, as would occur physiologically in preparation for parturition. As shown in Figures 3A,B, both hCG and estrogen promote a pro-NETotic state, as well as increased ROS production (Figure 3C) and elevations in citH3 protein (Figure 3D), important hallmarks of ensuing NETosis. Remarkably, progesterone had a pronounced anti-NETotic effect (Figures 3A,B), significantly diminishing the pro-NETotic influence of estrogen (Figures 3A,B). Though progesterone curbed ROS production, PAD4, and MPO protein expression induced by estrogen (Figure 3C; Figure S3A in Supplementary Material), citH3 levels remained unchanged (Figure 3D), suggesting a differential effect on signaling leading to NETosis. A detailed appraisal of the interaction between the two hormones in modulating NETosis is presented in Figure 3E, where it is evident that physiological concentrations of progesterone hindered the degree of NETosis attained by applications of estrogen alone. Progesterone was also effective in reducing NETosis triggered by the powerful stimulant PMA (Figure 3F; Figures S3B,C in Supplementary Material).

To further analyze the modulatory role of circulatory estrogen and progesterone on NETosis during pregnancy, we added specific inhibitors of the estrogen and progesterone receptors, fulvestrant and mifepristone (RU486), respectively, to pregnancy plasma samples and assayed NET formation (Figure 3G; Figures S3D,E in Supplementary Material). NETosis was reduced by the estrogen antagonist fulvestrant (Figure 3G; Figures S3D,E in Supplementary Material). This effect is presumably due to the unopposed action of progesterone, since gestational plasma samples contain an assortment of sex hormones. On the other hand, in samples treated with mifepristone, thereby inhibiting progesterone, NETosis was significantly increased. In this instance, the effect would be attributable to unopposed estrogen in the plasma sample.

These results show that NET formation is tightly regulated by the gestational hormonal milieu.

### Progesterone Confines NETosis of Neutrophils at an Advanced State of Priming

**Figure 4:**
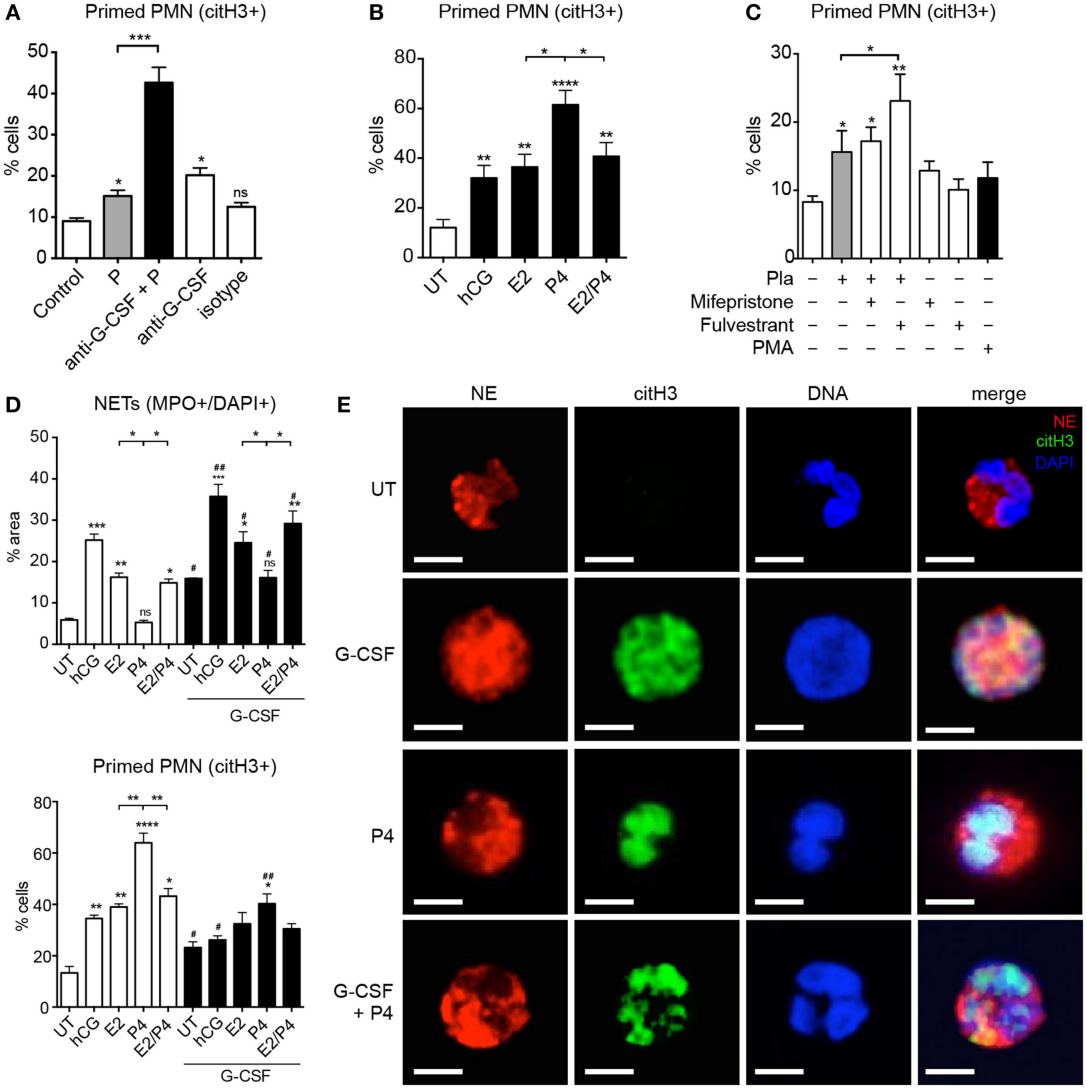
**Sex hormones differentially modulate G-CSF-driven neutrophil pro-NETotic priming**. **(A)** Morphometric analysis of the pro-NETotic primed (citH3+) neutrophils from healthy non-pregnant donors after *in vitro* incubation of control neutrophils with pooled pregnancy plasma with and without *in vitro* pretreatment with anti-G-CSF neutralizing antibody compared to the priming activity of control plasma. **(B)** Morphometric analysis of the pro-NETotic primed (citH3+) neutrophils from healthy non-pregnant donors after *in vitro* incubation of control neutrophils with physiologic pregnancy concentrations of hCG (50 IU/ml), E2 (20 ng/ml), P4 (50 ng/ml), and the combination of E2 and P4 for 3 h. **(C)** Morphometric analysis of the pro-NETotic primed citH3+ neutrophils from healthy non-pregnant donors under *in vitro* hormonal treatment and after *in vitro* preincubation of control neutrophils with the sex hormone inhibitors mifepristone and fulvestrant. **(D)** Morphometric analysis of the NETotic (MPO+/DAPI+) and pro-NETotic primed (citH3+) neutrophils from healthy non-pregnant donors without and with pretreatment with 12.5 ng/ml rhG-CSF for 1 h (lower panel) and stimulation with the gestational hormones hCG, E2, P4, and the combination of E2 and P4 for additional 2 h *in vitro*. **(E)** Fluorescent immunostaining and confocal microscopy for NE (red), citH3 (green), and DNA (blue) after a 3 h *in vitro* coculture of control neutrophils with rhG-CSF and addition of P4 compared to untreated control neutrophils. Scale bars: 10 μm. Data are presented as mean ± SEM. *P < 0.05, **P < 0.01, ***P < 0.001, and ****P < 0.0001 (one or two way ANOVA followed by Bonferroni’s multiple comparison post-test). All experiments were performed at least three times with consistent results.

During our experiments utilizing the blocking anti-G-CSF antibody in maternal plasma, we noted that such treatment not only led to a substantial reduction in NET formation (Figure 2E) but also to a significant increase in the number of primed citH3+ cells (Figure 4A). Since the anti-NETotic effect of G-CSF was greatest in samples collected close to term (Figure 2F), we speculated that this could be modulated by the pronounced anti-NETotic activity of progesterone (Figures 3A,B), concentrations of which are at their peak late in pregnancy (37). We indeed determined that the anti-NETotic effect of progesterone was associated with an increase in primed neutrophils, as identified by citH3 staining in the nucleus (Figure 4B). When neutrophils were exposed to gestational plasma samples treated with the estrogen antagonist fulvestrant (Figure 3G; Figures S3D,E in Supplementary Material), this was also evident.

Under physiological conditions, the combined action of G-CSF and estrogen appeared to limit the anti-NETotic effect of progesterone (Figure 4D; Figures S4A–C in Supplementary Material), while in the absence of estrogen, progesterone effectively limited NET formation initiated by G-CSF (Figure 4E). Once again, this anti-NETotic effect was coupled with a pronounced elevation in the number of primed neutrophils, as detected by citH3 staining (Figures 4C,D; Figure S4 in Supplementary Material).

Although the anti-NETotic action of progesterone appeared to involve reduced ROS production (Figure 3C), this mechanism in itself could not explain the high proportion of primed neutrophils characterized by citH3 staining (Figure 3A) or protein levels (Figure 3D). Rather, these data indicated that progesterone-treated neutrophils had undergone activation, involving nuclear translocation of PAD4 and histone citrullination, and yet were unable to proceed with NETosis.

Previous studies have indicated that cleavage of linker histones by NE present in the nucleus is required for the unfolding of chromatin and subsequent NETosis (18). Upon examining this facet by confocal microscopy, we noted diffuse nuclear staining for NE in cells treated with G-CSF (Figure 4E). This was in stark contrast to progesterone-treated cells, where nuclear translocation of NE did not occur (Figure 4E). Though these cells were citH3+, they also retained a distinctly lobulated nuclear structure, unlike the G-CSF treated cells, in which the chromatin had decondensed and dispersed into the cytoplasm.

### Further Aspects of Neutrophil Activity Modulated by Sex Hormones and G-CSF

**Figure 5:**
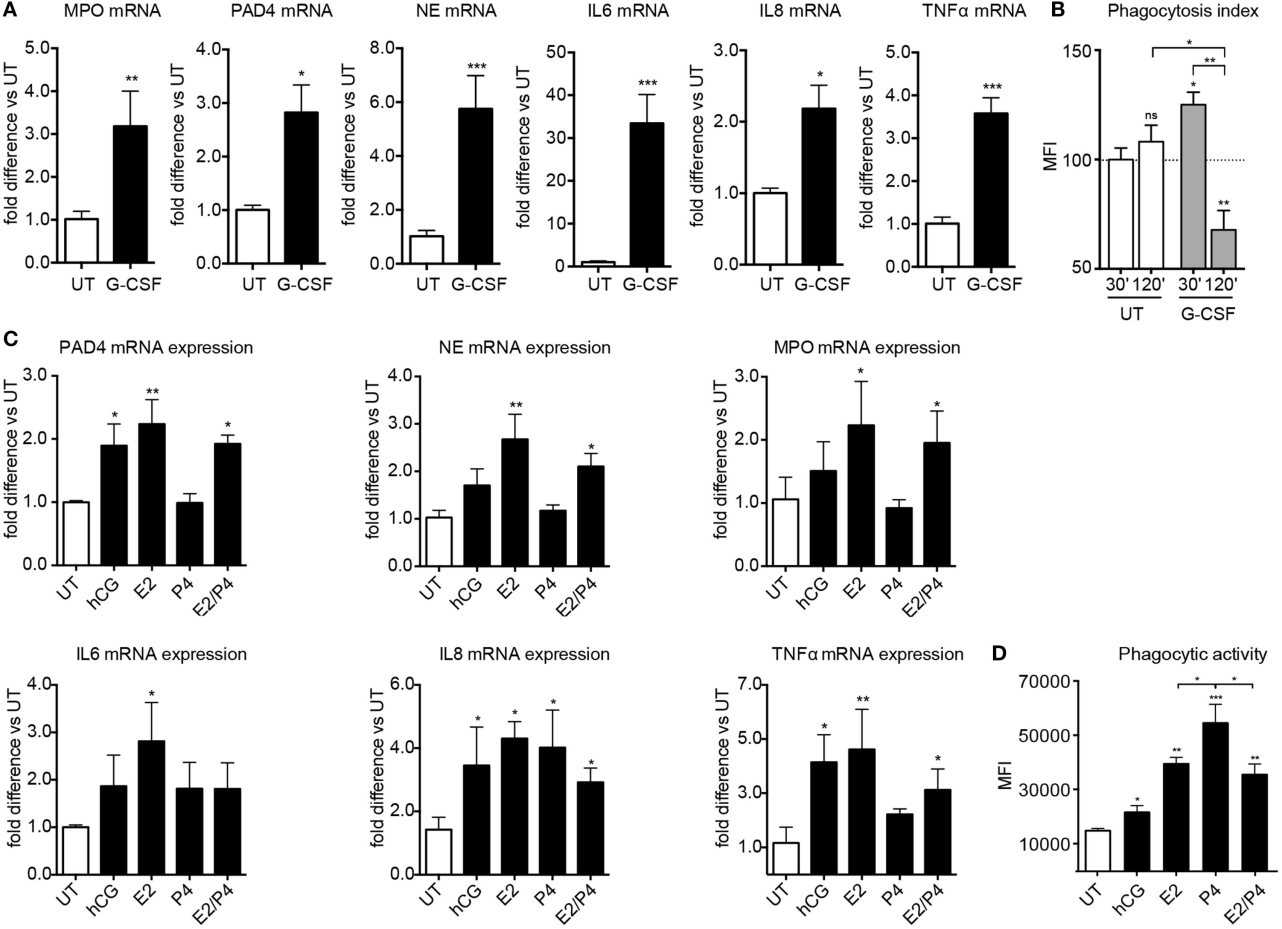
**Sex hormones and G-CSF regulate broader aspects of neutrophil effector functions**. **(A)** MPO, PAD4, NE, IL-6, IL-8, and TNFα gene expression analysis by Taqman qRT-PCR in RNA samples obtained from healthy female controls treated *in vitro* with 12.5 ng/ml rhG-CSF. **(B)** Phagocytic activity of neutrophils obtained from healthy female controls treated with 12.5 ng/ml rhG-CSF. **(C)** PAD4, NE, MPO, IL-6, IL-8, and TNF gene expression analysis by Taqman qRT-PCR in RNA samples obtained from healthy female control neutrophils treated *in vitro* with physiologic pregnancy concentrations of hCG (50 IU/ml), E2 (20 ng/ml), P4 (50 ng/ml), and the combination of E2 and P4 for 3 h. **(D)** Phagocytic activity in neutrophils from healthy control donors treated *in vitro* with physiologic pregnancy concentrations of hCG, E2, P4, and the combination of E2 and P4 for 3 h. Data are presented as mean ± SEM. *P < 0.05, **P < 0.01, ***P < 0.001, and ****P < 0.0001 (one or two way ANOVA followed by Bonferroni’s multiple comparison post-test). All experiments were performed at least six times with consistent results. MFI, mean fluorescence intensity; UT, untreated.

In agreement with previous reports, we observed that pregnancy was associated not only with the upregulation of NET related molecules, such as PAD4, MPO, and NE (Figure S5A in Supplementary Material), but also with an increased predisposition to phagocytosis (Figure S5B in Supplementary Material) and degranulation (Figure S5D in Supplementary Material). In parallel with the results for NETosis, both became more pronounced with advancing gestation. Accordingly, we studied the influence of G-CSF, estrogen, and progesterone on these aspects of neutrophil activity. Apart from promoting NETosis, G-CSF enhanced the expression of the granular proteins MPO and NE (Figure 5A) and promoted phagocytosis (Figure 5B). G-CSF also enhanced the expression of pro-inflammatory cytokines such as IL-6, IL-8, and TNF-α by neutrophils (Figure 5A).

The effect of estrogen and progesterone was more differentiated, with the former significantly enhancing the expression of MPO and NE, as well as that of IL-6 and TNF-α, in contrast to the rather dampened effect mediated by the latter (Figure 5C). Interestingly, both estrogen and progesterone trigger enhanced IL-8 expression, an important chemokine for neutrophil recruitment, degranulation, and NETosis (10, 39). This finding is important, since neutrophil expression of IL-8 progressively increases with advancing gestational age (Figure S5C in Supplementary Material), thereby indicating an important role of this pivotal cytokine in regulating neutrophil activity in pregnancy. In general, the influence of hCG was similar to that of estrogen, albeit to a lower level (Figures 5C,D). Furthermore, phagocytic activity was significantly more enhanced by progesterone treatment than by estrogen alone or by the combination of both (Figure 5D). These findings suggest that by blocking NETosis, progesterone may shift neutrophil antimicrobial activity toward other pathways, such as phagocytosis.

## Discussion

A crucial contribution to our understanding of PE, a highly inflammatory condition during gestation, was the seminal observation that it involved an excessive maternal inflammatory response to pregnancy (4). This was based on the finding that human pregnancy is associated with a subliminal inflammatory condition, characterized by enhanced neutrophil activation, which is most extreme in PE (3, 40, 41). The possible involvement of overtly activated neutrophils in the etiology of PE was further supported by the detection of large numbers of NETs in the intervillous space of affected placentae (10). Furthermore, data from animal models suggested that tissue-resident proangiogenic decidual neutrophils (42) and activation of circulatory neutrophils *via* the complement cascade may contribute to PE-like conditions or those associated with fetal loss (43, 44). The latter is supported by recent reports that antiphospholipid antibodies, which are frequently detected in cases with recurrent fetal loss, trigger NETosis [reviewed in Ref. (7)].

Unfortunately, the prominent focus on the role of neutrophils in pathological conditions has yielded few advances in the knowledge of their behavior during normal pregnancy (7, 12). As an understanding of the NETotic response in normal pregnancy could provide better insight into pathological subversion, it was the focus of our current study. Our data indicate that (i) circulatory neutrophils from pregnant women exhibit an enhanced pro-NETotic response, which increases with advancing gestation; (ii) pro-NETotic priming is mediated largely by G-CSF; (iii) NETosis is modulated in an opposing fashion by estrogen and progesterone; and (iv) in that progesterone locks neutrophils in a highly pro-NETotic state by hindering NE migration to the nucleus. Since neutrophil NETs were originally described as an antimicrobial mechanism (39), our data suggest that this operative arm of the innate immune response is highly proactive in human pregnancy. In this manner, by being in a highly primed pro-NETotic state, such pre-activated neutrophils could react immediately to a threat by pathogens.

Early in gestation, enhanced NETosis seems to be largely driven by hCG, which plays a pivotal role during this phase of pregnancy, with a minimal contribution by G-CSF. Interestingly, *in vitro* application of hCG leads to a significant increase in PAD4 expression, without a major increment in citH3 levels. Furthermore, ROS production, a further key element in the NETotic cascade, was not significantly elevated by hCG treatment, underlining a possible ROS-independent mechanism driving the generation of NETs as described recently (45). As gestation advances, it becomes apparent that a discrete interaction between G-CSF and steroid sex hormones modulates pro-NETotic activity. In this regard, elevated concentrations of G-CSF during pregnancy not only serve to increase the pool of circulatory neutrophils but also to promote a primed pro-NETotic phenotype. This is achieved by triggering increased PAD4 expression and concomitant histone H3 citrullination, essential steps in the NETotic signaling cascade (21, 22).

The most intriguing aspect of our analysis was the antagonistic interplay between estrogen and progesterone in regulating NETosis. While a host of literature supports the contrary influence of progesterone on estrogen-mediated effects (46), our observation is novel in that progesterone appears to exploit key signaling events to prevent the extrusion of NETs. Previous studies have shown that the triggering of NET formation requires several key steps, including ROS production, calcium mobilization, and activation of PKC and MAP kinases. On the other hand, untangling of chromatin, an *a priori* requirement for decondensation of the nuclear chromosomal DNA and its subsequent expulsion into the extracellular milieu, has been shown to involve citrullination of histones by PAD4 (21, 22), as well as the proteolytic clipping of histones (notably H4) by NE (18). Upon activation of neutrophils, ROS production leads to the release of NE from azurophilic granules into the cytosol. It then translocates to the nucleus, where it cleaves histones to extensively decondense chromatin (18). MPO, also originating from the azurophilic granules, consumes H_2_O_2_ to generate HOCl and other oxidants and is required for translocation of NE to the nucleus during NETosis by regulating actin dynamics (18, 23). Additionally, phagocytosis seems to negatively regulate NETosis (23). Our data indicate that progesterone downregulates ROS production in neutrophils, which is in concordance to previous studies showing that progesterone is effective in reducing ROS and NO generation driven by estradiol (47, 48). The observed inhibition of ROS production occurs most probably in favor of a significant shift towards the involvement of calcium signaling and PAD4 mobilization, which most probably promotes phagocytosis rather than degranulation or formation of NETs (20). The latter might lead to the excessive histone citrullination detected both *in vitro* and *ex vivo*, needed for the formation of NETs under certain stimuli, bringing NE and MPO into the nucleus. This translocation signifies the crucial event leading to the extrusion of NETs from the activated neutrophils. In pregnancy, the antagonistic effect of progesterone is manifested by hindering translocation of NE to nucleus, thereby preventing terminal nuclear decondensation and disruption of the nuclear membrane, with subsequent intermingling between nuclear and cytoplasmic contents. Progesterone seems to permit histone citrullination by PAD4, thus effectively locking neutrophils in a highly primed pro-NETotic state, till triggered by a second stimulus that overcomes this transitory impediment.

Our data also extend upon previous reports indicating that circulatory neutrophils exhibit an increased propensity for degranulation and phagocytosis during pregnancy (4, 9), shedding new light on their regulation by the action of G-CSF and sex hormones. In this regard, phagocytosis is enhanced by treatment with G-CSF. However, this is most pronounced in short-term cultures of 30 min, as it is drastically diminished after 2 h. In the instance of the hormones, the enhancement of phagocytosis is slower than that for G-CSF, requiring an incubation period of 2 h for maximal effect. Of interest is that phagocytosis was most markedly affected by progesterone, while it is reduced when using estrogen in combination with progesterone under physiological concentrations. This suggests that progesterone may promote antibacterial activity involving phagocytosis rather than NETosis, while in combination with estrogen, this effect is diminished. It is also clear that the interaction between G-CSF and sex hormones influences a broader scope of neutrophil activity, including expression of key components of the NETotic cascade, such as PAD4, NE, and MPO, as well as pro-inflammatory cytokines and chemokines, such as TNF-α and IL-8. The interplay between these diverse facets may be important in ensuring that neutrophils contribute to an effective innate immune response during pregnancy.

In the same token, it is feasible that aberrances in this system may contribute to the pathogenesis of severe pregnancy-related conditions such as PE. This hypothesis is supported by observations that PE is associated with elevated levels of hCG (49), G-CSF (50), as well as indications of reduced progesterone levels (51), while therapy with synthetic P4 ameliorates PE symptoms in an animal model (52). It is therefore possible that such an imbalance may trigger an enhanced pro-NETotic response, which is exacerbated by the presence of inflammatory placental micro-debris (4), abundant in this condition. On the other hand, our data may provide a novel insight into autoimmune conditions such as SLE, which is associated with reduced levels of progesterone, both during the menstrual cycle, as well as during pregnancy (53). Since NETosis is altered in cases with SLE (54), it is possible that the negative feedback loop hindering NET formation provided by progesterone contributes to this phenomenon. As estrogen levels are normal in these patients, the altered ratio between the two sex steroid hormones could lead to a more facile triggering of the NETotic cascade. It is, hence, possible that this phenomenon may contribute to the PE-like symptoms frequently observed in pregnant women affected by SLE.

**Figure 6:**
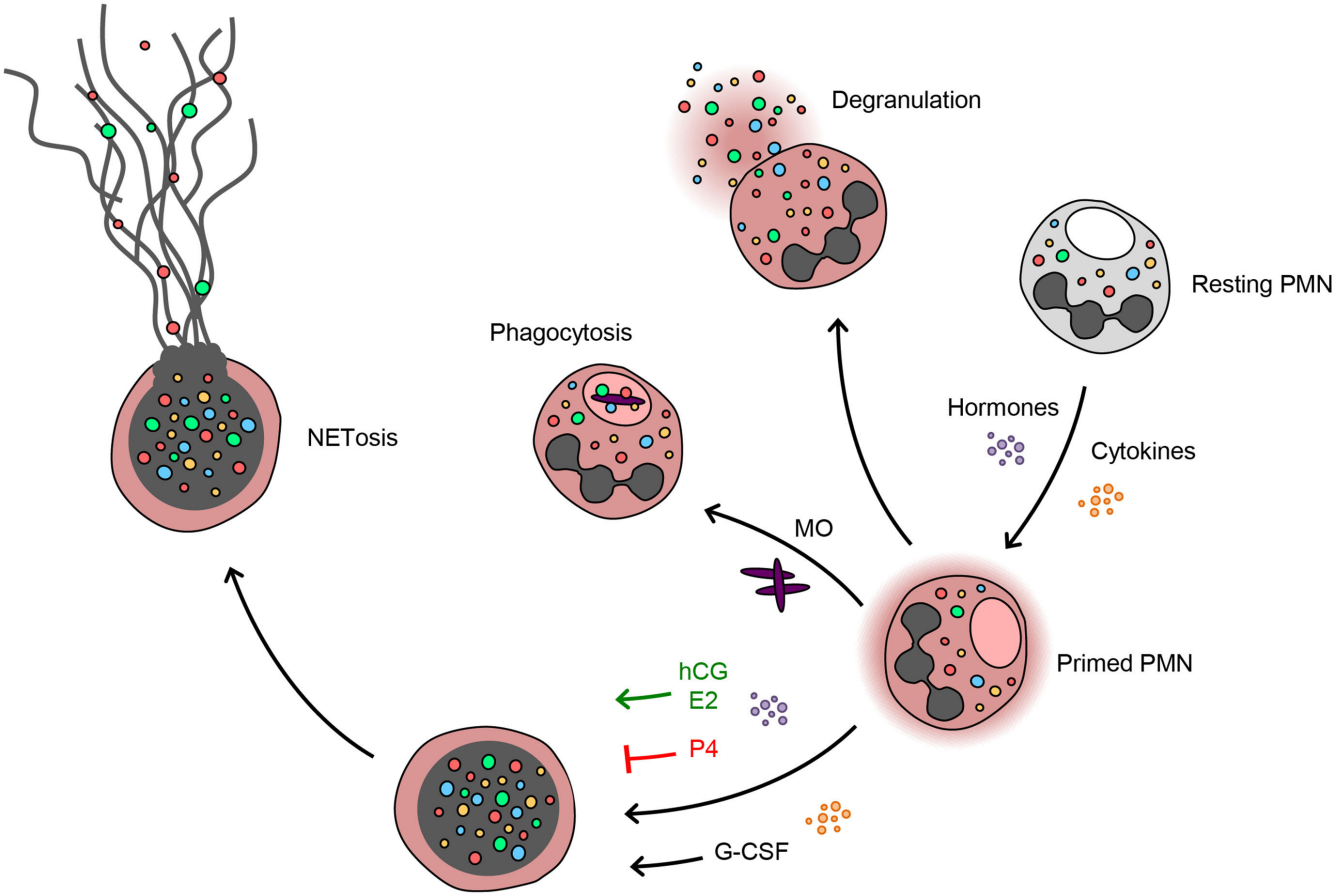
**Progesterone antagonizes the estrogen and G-CSF-driven neutrophil extracellular trap formation during pregnancy**. Neutrophils during pregnancy lie under the increased influence of cytokines, e.g., G-CSF, and sex hormones. This specific milieu appears to poise the neutrophils in a pro-NETotic primed state. Depending on the stimulus, neutrophils react by phagocytosis or degranulation. When a different NETotic stimulus is present, such as systemic infection or excessive placentally derived plasma microparticles (MP) in preeclampsia (10), primed neutrophils react with overt NET release. Pro-NETotic combinations of hormones and cytokines are given in green, inhibitory combinations are given in red.

In summary, the present study demonstrates that during pregnancy neutrophils exhibit a pro-NETotic primed state and enhanced propensity to release NETs. This activity is modulated at several key levels. First, G-CSF seems to be a major signal for neutrophil release from the bone marrow throughout gestation, providing an important mechanism increasing the levels of circulating neutrophils and promoting pro-NETotic priming. Second, the degree of NET formation seems to be finely tuned by the balance of the sex steroid hormones, which are produced exclusively by the placenta and reach their peak concentrations toward term. Finally, NETs are released under the presence of specific gestational secondary stimuli (Figure 6).

Our findings regarding neutrophil responses during normal pregnancy provide new insight concerning gestational and hormone-driven pathologies, since neutrophil recruitment, activation, and NET release could be associated with excessive endothelial and placental injury. Based on these premises, we suggest that the proportions of the sex steroid hormones unique to each individual condition, physiologic or pathologic, tune the innate immune system and neutrophils in particular into a specific functional mode of action. When this balance is lost or absent, neutrophils react with overt NET formation, which in turn leads to tissue injury. With regard to pregnancy, it is plausible that in extreme conditions of NETosis, such as shown previously in PE, these diverse features might become detrimental and promote a strong procoagulant state, possibly resulting in placental infarction.

## Author Contributions

SG and MS performed all experiments, except as noted below; CC performed part of the ELISA and immunoblot analysis; GS performed part of the cell isolations and IHC stainings; FG performed part of the cell isolations; IH and OL provided advice for and contributed to the recruitment of the sample donors; SG and SH devised and directed the study; SR provided advice and contributed to the revision of the manuscript; and SG, PH, and SH wrote the manuscript.

## Conflict of Interest Statement

The authors declare that the research was conducted in the absence of any commercial or financial relationships that could be construed as a potential conflict of interest.

## Footnotes

^1^www.graphpad.com.

^2^www.eudox.ch.

## Abbreviations

citH3, citrullinated histone H3; DAPI, 4′,6-diamidino-2-phenylindole; E2, β-estradiol; E3, estriol; EIA, enzyme immunoassay; G-CSF, granulocyte colony-stimulating factor; hCG, human chorionic gonadotropin; MPO, myeloperoxidase; NADPH, nicotinamide adenine dinucleotide phosphate oxidase; NE, neutrophil elastase; NET, neutrophil extracellular trap; P4, progesterone; PAD4, peptidyl arginine deiminase IV; PMA, phorbol myristate acetate; RA, rheumatoid arthritis; ROS, reactive oxygen species; SLE, systemic lupus erythematosus.

## References

[1] Arck PC Hecher K. Fetomaternal immune cross-talk and its consequences for maternal and offspring’s health. Nat Med (2013) 19:548–56. 10.1038/nm.3160 23652115

[2] Mor G Cardenas I. The immune system in pregnancy: a unique complexity. Am J Reprod Immunol (2010) 63:425–33. 10.1111/j.1600-0897.2010.00836.x 20367629 PMC3025805

[3] Sacks G Sargent I Redman C An innate view of human pregnancy. Immunol Today (1999) 20:114–8. 10.1016/S0167-5699(98)01393-0 10203701

[4] Redman CW Sacks GP Sargent IL. Preeclampsia: an excessive maternal inflammatory response to pregnancy. Am J Obstet Gynecol (1999) 180:499–506. 10.1016/S0002-9378(99)70239-5 9988826

[5] Girardi G Berman J Redecha P Spruce L Thurman JM Kraus D Complement C5a receptors and neutrophils mediate fetal injury in the antiphospholipid syndrome. J Clin Invest (2003) 112:1644–54. 10.1172/JCI200318817 14660741 PMC281643

[6] Soh MC Nelson-Piercy C. High-risk pregnancy and the rheumatologist. Rheumatology (Oxford) (2015) 54:572–87. 10.1093/rheumatology/keu394 25477056

[7] Giaglis S Stoikou M Grimolizzi F Subramanian BY van Breda SV Hoesli I Neutrophil migration into the placenta: good, bad or deadly? Cell Adh Migr (2016) 10(1–2):208–25. 10.1080/19336918.2016.1148866 26933824 PMC4853040

[8] Sur Chowdhury C Hahn S Hasler P Hoesli I Lapaire O Giaglis S. Elevated levels of total cell-free DNA in maternal serum samples arise from the generation of neutrophil extracellular traps. Fetal Diagn Ther (2016) 40. 10.1159/000444853 26998969

[9] Sacks GP Studena K Sargent K Redman CW. Normal pregnancy and preeclampsia both produce inflammatory changes in peripheral blood leukocytes akin to those of sepsis. Am J Obstet Gynecol (1998) 179:80–6. 10.1016/S0002-9378(98)70254-6 9704769

[10] Gupta AK Hasler P Holzgreve W Gebhardt S Hahn S. Induction of neutrophil extracellular DNA lattices by placental microparticles and IL-8 and their presence in preeclampsia. Hum Immunol (2005) 66:1146–54. 10.1016/j.humimm.2005.11.003 16571415

[11] Marder W Knight JS Kaplan MJ Somers EC Zhang X O’Dell AA Placental histology and neutrophil extracellular traps in lupus and pre-eclampsia pregnancies. Lupus Sci Med (2016) 3:e000134. 10.1136/lupus-2015-000134 27158525 PMC4854113

[12] Hahn S Giaglis S Hoesli I Hasler P. Neutrophil NETs in reproduction: from infertility to preeclampsia and the possibility of fetal loss. Front Immunol (2012) 3:362. 10.3389/fimmu.2012.00362 23205021 PMC3506920

[13] Borregaard N. Neutrophils, from marrow to microbes. Immunity (2010) 33:657–70. 10.1016/j.immuni.2010.11.011 21094463

[14] Mocsai A. Diverse novel functions of neutrophils in immunity, inflammation, and beyond. J Exp Med (2013) 210:1283–99. 10.1084/jem.20122220 23825232 PMC3698517

[15] Hahn S Giaglis S Chowdury CS Hosli I Hasler P. Modulation of neutrophil NETosis: interplay between infectious agents and underlying host physiology. Semin Immunopathol (2013) 35(4):439–53. 10.1007/s00281-013-0380-x 23649713 PMC3685704

[16] Sorensen OE Borregaard N. Neutrophil extracellular traps – the dark side of neutrophils. J Clin Invest (2016) 126:1612–20. 10.1172/JCI84538 27135878 PMC4855925

[17] Fuchs TA Abed U Goosmann C Hurwitz R Schulze I Wahn V Novel cell death program leads to neutrophil extracellular traps. J Cell Biol (2007) 176:231–41. 10.1083/jcb.200606027 17210947 PMC2063942

[18] Papayannopoulos V Metzler KD Hakkim A Zychlinsky A. Neutrophil elastase and myeloperoxidase regulate the formation of neutrophil extracellular traps. J Cell Biol (2010) 191:677–91. 10.1083/jcb.201006052 20974816 PMC3003309

[19] Leshner M Wang S Lewis C Zheng H Chen XA Santy L PAD4 mediated histone hypercitrullination induces heterochromatin decondensation and chromatin unfolding to form neutrophil extracellular trap-like structures. Front Immunol (2012) 3:307. 10.3389/fimmu.2012.00307 23060885 PMC3463874

[20] Gupta A Giaglis S Hasler P Hahn S. Efficient neutrophil extracellular trap induction requires mobilization of both intracellular and extracellular calcium pools and is modulated by cyclosporine A. Plos One (2014) 9(5):e97088. 10.1371/journal.pone.0097088 24819773 PMC4018253

[21] Wang Y Li M Stadler S Correll S Li P Wang D Histone hypercitrullination mediates chromatin decondensation and neutrophil extracellular trap formation. J Cell Biol (2009) 184:205–13. 10.1083/jcb.200806072 19153223 PMC2654299

[22] Martinod K Demers M Fuchs TA Wong SL Brill A Gallant M Neutrophil histone modification by peptidylarginine deiminase 4 is critical for deep vein thrombosis in mice. Proc Natl Acad Sci U S A (2013) 110:8674–9. 10.1073/pnas.1301059110 23650392 PMC3666755

[23] Metzler KD Goosmann C Lubojemska A Zychlinsky A Papayannopoulos V. A myeloperoxidase-containing complex regulates neutrophil elastase release and actin dynamics during NETosis. Cell Rep (2014) 8:883–96. 10.1016/j.celrep.2014.06.044 25066128 PMC4471680

[24] Zhong XY von Muhlenen I Li Y Kang A Gupta AK Tyndall A Increased concentrations of antibody-bound circulatory cell-free DNA in rheumatoid arthritis. Clin Chem (2007) 53:1609–14. 10.1373/clinchem.2006.084509 17712000

[25] Sur Chowdhury C Giaglis S Walker UA Buser A Hahn S Hasler P. Enhanced neutrophil extracellular trap generation in rheumatoid arthritis: analysis of underlying signal transduction pathways and potential diagnostic utility. Arthritis Res Ther (2014) 16:R122. 10.1186/ar4579 24928093 PMC4229860

[26] Gupta AK Joshi MB Philippova M Erne P Hasler P Hahn S Activated endothelial cells induce neutrophil extracellular traps and are susceptible to NETosis-mediated cell death. FEBS Lett (2010) 584:3193–7. 10.1016/j.febslet.2010.06.006 20541553

[27] Kessenbrock K Krumbholz M Schonermarck U Back W Gross WL Werb Z Netting neutrophils in autoimmune small-vessel vasculitis. Nat Med (2009) 15:623–5. 10.1038/nm.1959 19448636 PMC2760083

[28] LeBel CP Ischiropoulos H Bondy SC. Evaluation of the probe 2′,7′-dichlorofluorescin as an indicator of reactive oxygen species formation and oxidative stress. Chem Res Toxicol (1992) 5:227–31. 10.1021/tx00026a012 1322737

[29] Kirchner T Moller S Klinger M Solbach W Laskay T Behnen M. The impact of various reactive oxygen species on the formation of neutrophil extracellular traps. Mediators Inflamm (2012) 2012:849136. 10.1155/2012/849136 22481865 PMC3317033

[30] Brechard S Bueb JL Tschirhart EJ. Interleukin-8 primes oxidative burst in neutrophil-like HL-60 through changes in cytosolic calcium. Cell Calcium (2005) 37:531–40. 10.1016/j.ceca.2005.01.019 15862344

[31] Brinkmann V Goosmann C Kuhn LI Zychlinsky A Automatic quantification of in vitro NET formation. Front Immunol (2013) 3:413 10.3389/fimmu.2012.00413 23316198 PMC3540390

[32] Bustin SA Benes V Garson JA Hellemans J Huggett J Kubista M The MIQE guidelines: minimum information for publication of quantitative real-time PCR experiments. Clin Chem (2009) 55:611–22. 10.1373/clinchem.2008.112797 19246619

[33] Livak KJ Schmittgen TD. Analysis of relative gene expression data using real-time quantitative PCR and the 2(-Delta Delta C(T)) Method. Methods (2001) 25:402–8. 10.1006/meth.2001.1262 11846609

[34] Shechter D Dormann HL Allis CD Hake SB. Extraction, purification and analysis of histones. Nat Protoc (2007) 2:1445–57. 10.1038/nprot.2007.202 17545981

[35] Demers M Krause DS Schatzberg D Martinod K Voorhees JR Fuchs TA Cancers predispose neutrophils to release extracellular DNA traps that contribute to cancer-associated thrombosis. Proc Natl Acad Sci U S A (2012) 109:13076–81. 10.1073/pnas.1200419109 22826226 PMC3420209

[36] Braunstein GD Rasor J Danzer H Adler D Wade ME. Serum human chorionic gonadotropin levels throughout normal pregnancy. Am J Obstet Gynecol (1976) 126:678–81. 10.1016/0002-9378(76)90518-4 984142

[37] Johansson ED Plasma levels of progesterone in pregnancy measured by a rapid competitive protein binding technique. Acta Endocrinol (Copenh) (1969) 61:607–17. 5409083 10.1530/acta.0.0610607

[38] Levitz M Young BK Estrogens in pregnancy. Vitam Horm (1977) 35:109–47. 10.1016/S0083-6729(08)60522-1 343361

[39] Brinkmann V Reichard U Goosmann C Fauler B Uhlemann Y Weiss DS Neutrophil extracellular traps kill bacteria. Science (2004) 303:1532–5. 10.1126/science.1092385 15001782

[40] Kindzelskii AL Huang JB Chaiworapongsa T Fahmy RM Kim YM Romero R Pregnancy alters glucose-6-phosphate dehydrogenase trafficking, cell metabolism, and oxidant release of maternal neutrophils. J Clin Invest (2002) 110:1801–11. 10.1172/JCI200215973 12488430 PMC151652

[41] Osorio Y Bonilla DL Peniche AG Melby PC Travi BL. Pregnancy enhances the innate immune response in experimental cutaneous leishmaniasis through hormone-modulated nitric oxide production. J Leukoc Biol (2008) 83:1413–22. 10.1189/jlb.0207130 18347075

[42] Amsalem H Kwan M Hazan A Zhang J Jones RL Whittle W Identification of a novel neutrophil population: proangiogenic granulocytes in second-trimester human decidua. J Immunol (2014) 193:3070–9. 10.4049/jimmunol.1303117 25135830

[43] Ahmed A Singh J Khan Y Seshan SV Girardi G. A new mouse model to explore therapies for preeclampsia. PLoS One (2010) 5:e13663. 10.1371/journal.pone.0013663 21048973 PMC2965104

[44] Hahn S Lapaire O Than NG. Biomarker development for presymptomatic molecular diagnosis of preeclampsia: feasible, useful or even unnecessary? Expert Rev Mol Diagn (2015) 15:617–29. 10.1586/14737159.2015.1025757 25774007 PMC4673513

[45] Rochael NC Guimaraes-Costa AB Nascimento MT Desouza-Vieira TS Oliveira MP Garcia E Souza LF Classical ROS-dependent and early/rapid ROS-independent release of neutrophil extracellular traps triggered by leishmania parasites. Sci Rep (2015) 5:18302. 10.1038/srep18302 26673780 PMC4682131

[46] Wira CR Rodriguez-Garcia M Patel MV. The role of sex hormones in immune protection of the female reproductive tract. Nat Rev Immunol (2015) 15:217–30. 10.1038/nri3819 25743222 PMC4716657

[47] Itagaki T Shimizu X Cheng Y Yuan A Oshio K Tamaki H Opposing effects of oestradiol and progesterone on intracellular pathways and activation processes in the oxidative stress induced activation of cultured rat hepatic stellate cells. Gut (2005) 54:1782–9. 10.1136/gut.2005.053278 16284289 PMC1774809

[48] Maleki J Nourbakhsh M Shabani M Korani M Nourazarian SM Ostadali Dahaghi MR 17beta-Estradiol stimulates generation of reactive species oxygen and nitric oxide in ovarian adenocarcinoma cells (OVCAR 3). Iran J Cancer Prev (2015) 8(3):e2332 10.17795/ijcp2332 26413252 PMC4581366

[49] Lapaire O Grill S Lalevee S Kolla V Hosli I Hahn S. Microarray screening for novel preeclampsia biomarker candidates. Fetal Diagn Ther (2012) 31:147–53. 10.1159/000337325 22472943

[50] Matsubara K Ochi H Kitagawa H Yamanaka K Kusanagi Y Ito M. Concentrations of serum granulocyte-colony-stimulating factor in normal pregnancy and preeclampsia. Hypertens Pregnancy (1999) 18:95–106. 10.3109/10641959909009614 10464003

[51] Uddin MN Horvat D Jones RO Beeram MR Zawieja DC Perger L Suppression of aldosterone and progesterone in preeclampsia. J Matern Fetal Neonatal Med (2014) 28(11):1–6. 10.3109/14767058.2014.951627 25164552

[52] Amaral LM Cornelius DC Harmon A Moseley J Martin JN Jr Lamarca B. 17-hydroxyprogesterone caproate significantly improves clinical characteristics of preeclampsia in the reduced uterine perfusion pressure rat model. Hypertension (2015) 65:225–31. 10.1161/HYPERTENSIONAHA.114.04484 25368030 PMC4350787

[53] Hughes GC. Progesterone and autoimmune disease. Autoimmun Rev (2012) 11:A502–14. 10.1016/j.autrev.2011.12.003 22193289 PMC3431799

[54] Knight JS Kaplan MJ. Lupus neutrophils: ‘NET’ gain in understanding lupus pathogenesis. Curr Opin Rheumatol (2012) 24:441–50. 10.1097/BOR.0b013e3283546703 22617827

