# Supplementary material for: Multimodal Regulation of NET Formation in Pregnancy: Progesterone Antagonizes the Pro-NETotic Effect of Estrogen and G-CSF
Source: Front Immunol. 2016 Dec 5;7:565. doi: 10.3389/fimmu.2016.00565 (PMC5136684; doi:10.3389/fimmu.2016.00565)
Supplement: Supplementary file 1 [file Table_S1.PDF]

	Non-pregnant donors	Pregnant donors	P
n	40	45	na
		IT: 15	
		IIT: 25	
		IIIT: 35	
Maternal age (years)	32.7 (23-46)	33.9 (26-41)	ns
Gestational age (weeks)	na	IT: 12.5 (12-13)	na
		IIT: 24.7 (22-26)	
		IIIT: 39.1 (37-41)	

Values expressed as mean ± SE with minimum-maximum, where applicable; IT, first trimester; IIT, second trimester, IIIT, third trimester ns, not significant; na, not applicable.

Table S1. Demographic characteristics of the study cohort, i.e. women during the three trimesters of pregnancy and non-pregnant healthy controls.
