# Supplementary material for: Multimodal Regulation of NET Formation in Pregnancy: Progesterone Antagonizes the Pro-NETotic Effect of Estrogen and G-CSF
Source: Front Immunol. 2016 Dec 5;7:565. doi: 10.3389/fimmu.2016.00565 (PMC5136684; doi:10.3389/fimmu.2016.00565)
Supplement: Supplementary file 2 [file Video_S1.PPTX]

## Slide 1
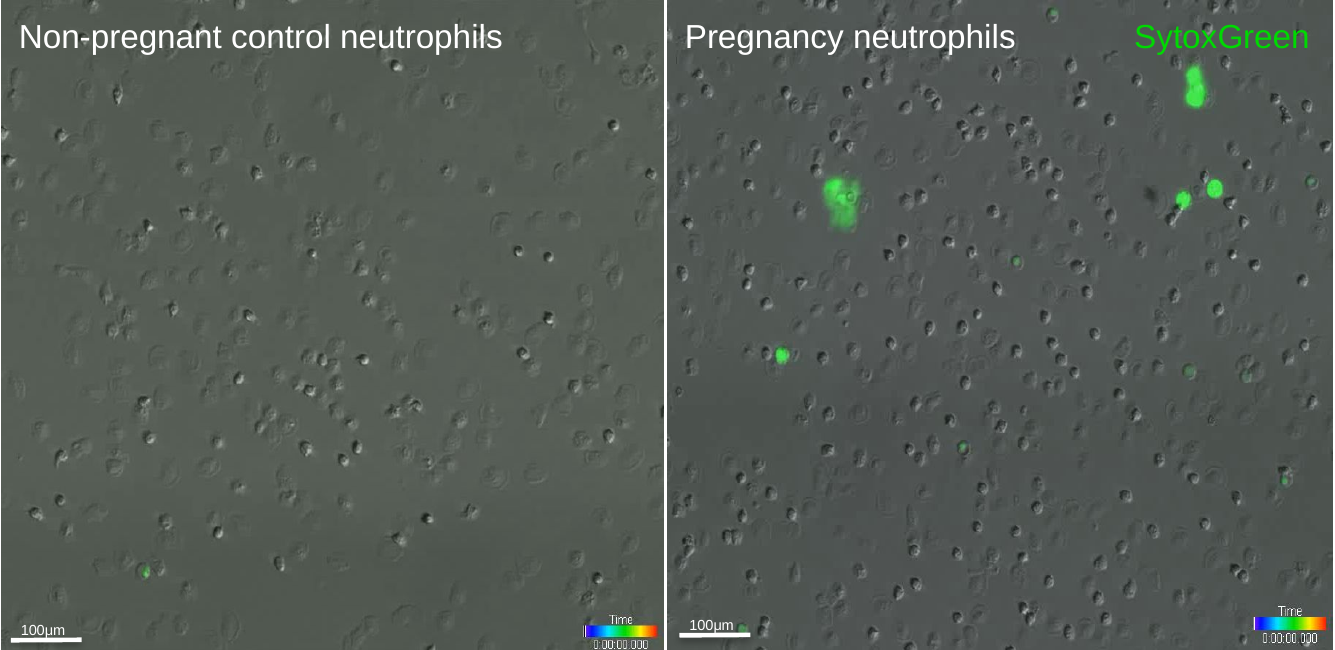

Non-pregnant control neutrophils
Pregnancy neutrophils
SytoxGreen
100μm
100μm

## Slide 2
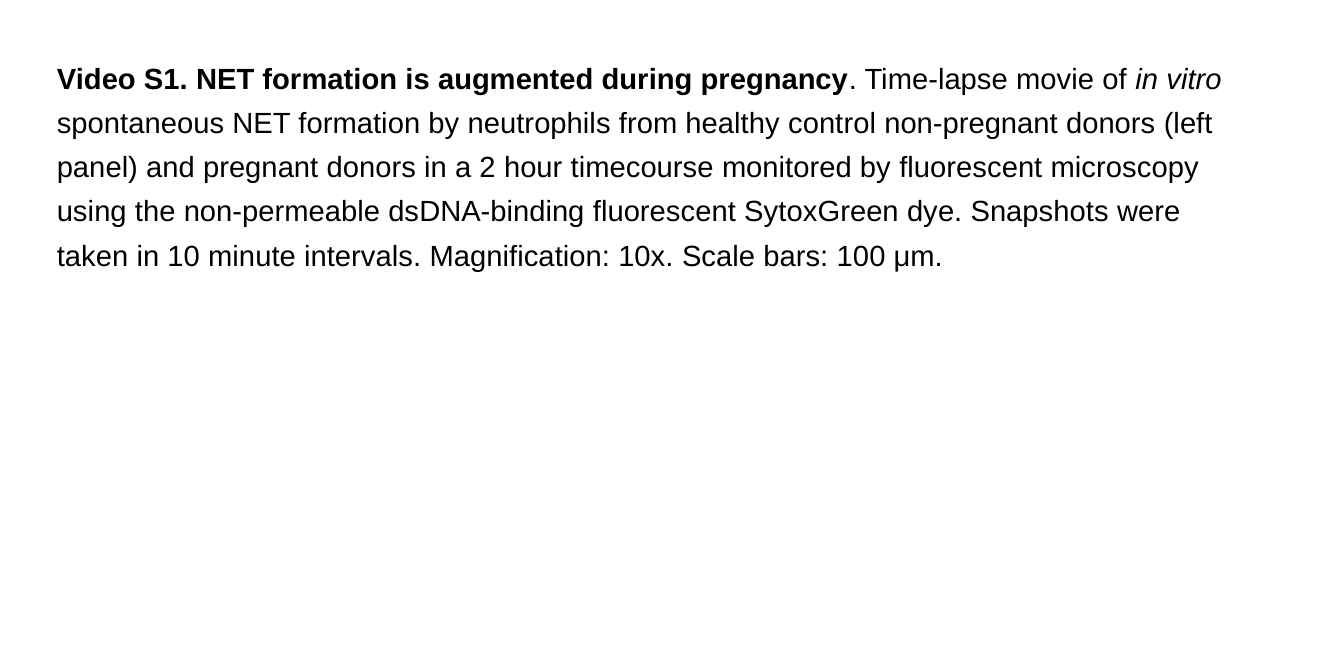

Video S1. NET formation is augmented during pregnancy. Time-lapse movie of in vitro spontaneous NET formation by neutrophils from healthy control non-pregnant donors (left panel) and pregnant donors in a 2 hour timecourse monitored by fluorescent microscopy using the non-permeable dsDNA-binding fluorescent SytoxGreen dye. Snapshots were taken in 10 minute intervals. Magnification: 10x. Scale bars: 100 μm.
