# Supplementary material for: Multimodal Regulation of NET Formation in Pregnancy: Progesterone Antagonizes the Pro-NETotic Effect of Estrogen and G-CSF
Source: Front Immunol. 2016 Dec 5;7:565. doi: 10.3389/fimmu.2016.00565 (PMC5136684; doi:10.3389/fimmu.2016.00565)
Supplement: Supplementary file 3 [file Figure_S1.PDF]

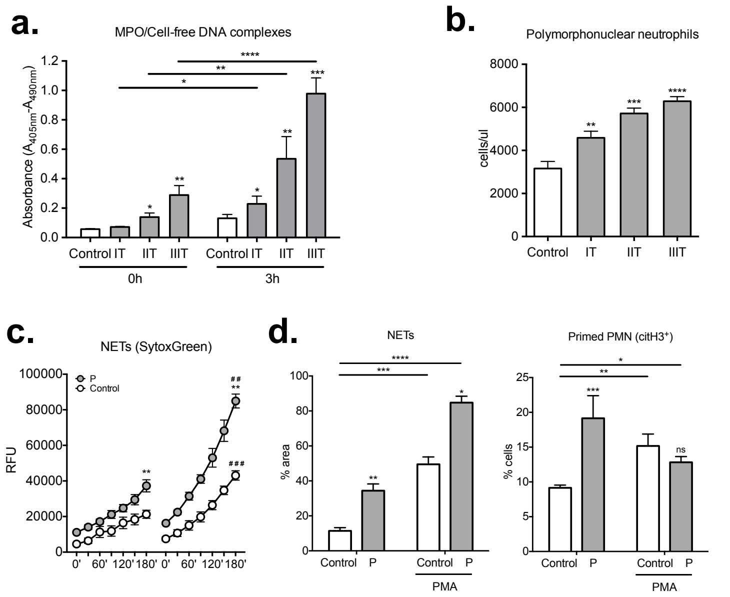

Fig. S1. Neutrophil pro-NETotic priming and NET formation are upregulated during pregnancy. (**a**) Quantification of NET-associated MPO/DNA complex levels in culture supernatants of neutrophils from healthy controls and pregnant donors at baseline (0h) and at 3 hours (3h) during the three trimesters of pregnancy. (**b**) Bar graph depicting the differential neutrophil count in blood samples obtained from healthy female controls during the three trimesters of pregnancy compared to non-pregnant control blood donors. (**c**) *In vitro* spontaneous NET release monitored in a 3 hour time course by dsDNA-binding fluorescent SytoxGreen dye after application of PMA as secondary stimulus. (**d**) Morphometric analysis of the NETotic (MPO+) and pro-NETotic primed (citH3+) neutrophils from healthy donors and donors during pregnancy after application of PMA as secondary stimulus. Data are presented as mean ± SEM. *P < 0.05, **P < 0.01, ***P < 0.001, ****P < 0.0001 (one way ANOVA followed by Bonferroni's multiple comparison post-test). All experiments were performed at least 6 times with consistent results. IT, first trimester; IIT, second trimester; IIIT, third trimester; RFU, relative fluorescence units; P, pregnancy.
