# Supplementary material for: Multimodal Regulation of NET Formation in Pregnancy: Progesterone Antagonizes the Pro-NETotic Effect of Estrogen and G-CSF
Source: Front Immunol. 2016 Dec 5;7:565. doi: 10.3389/fimmu.2016.00565 (PMC5136684; doi:10.3389/fimmu.2016.00565)
Supplement: Supplementary file 4 [file Figure_S2.PDF]

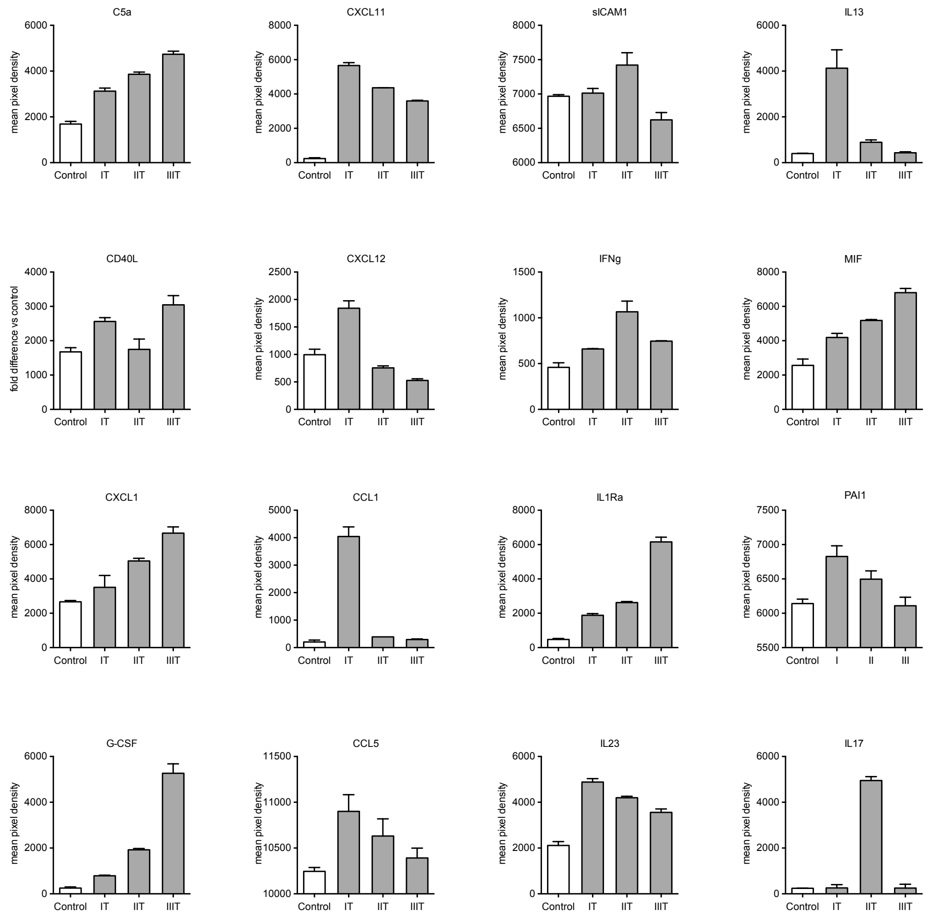

Fig. S2. Pregnancy changes in plasma concentrations of G-CSF determines neutrophil pro-NETotic priming during gestation. Serum levels of C5a, CD40L, CXCL1, G-CSF, CXCL11, CXCL12, CCL1, CCL5, cICAM1, IFNγ , IL1Ra, IL13, IL17, IL23, MIF and PAI1 by Human Cytokine Array analysis (R&D Systems). IT, first trimester; IIT, second trimester; IIIT, third trimester; RFU, relative fluorescence units.
