# Supplementary material for: Multimodal Regulation of NET Formation in Pregnancy: Progesterone Antagonizes the Pro-NETotic Effect of Estrogen and G-CSF
Source: Front Immunol. 2016 Dec 5;7:565. doi: 10.3389/fimmu.2016.00565 (PMC5136684; doi:10.3389/fimmu.2016.00565)
Supplement: Supplementary file 5 [file Figure_S3.PDF]

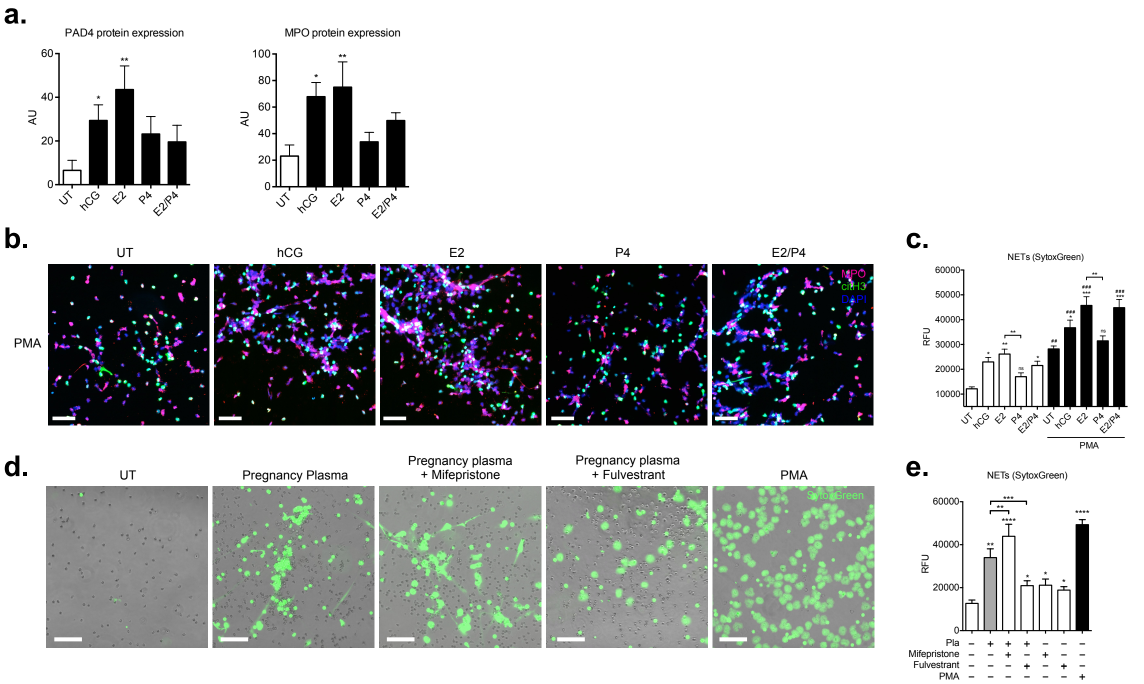

Fig. S3. Neutrophil *in vitro* pro-NETotic priming is regulated by pregnancy hormones. (**a**) Western blot and densitometric analysis of PAD4 and MPO normalized to beta-actin protein expression levels in neutrophil lysates from healthy female controls after incubation with physiologic pregnancy concentrations of the sex hormones hCG (50 IU/ml), E2 (20 ng/ml), P4 (50 ng/ml) and the combination of 20 ng/ml E2 and 50 ng/ml for 2 hours *in vitro*. (**b**) *In vitro* spontaneous NET release monitored microscopically over a 3 hour time course by fluorescent immunostaining for MPO (red), citH3 (green) and DNA (blue) after 1 hour pretreatment with physiologic concentrations of hCG, E2, P4 and E2/P4 and stimulation with the NET-inducing agent PMA as a second hit for additional 2 hours. Magnification: 20x; Scale bars: 50 µm. (**c**) Quantification of extracellular DNA release of gestational hormone treated control neutrophils after addition of PMA by fluorimetry. (**d**) Detection of *in vitro* spontaneous NET formation of neutrophils from healthy controls co-cultured with pregnancy plasma pretreated with mifepristone and fulvestrant over a 3 hour time course by fluorescent microscopy using dsDNA-binding fluorescent SytoxGreen dye. Magnification: 10x. Scale bars: 100 µm. (**e**) Fluorimetric quantification of extracellular DNA release of pregnancy plasma treated control neutrophils and preincubation with the sex hormone inhibitors mifepristone and fulvestrant. Data are presented as mean ± SEM. *P < 0.05, **P < 0.01, ***P < 0.001, ****P < 0.0001 (one way ANOVA followed by Bonferroni's multiple comparison post-test). All experiments were performed at least 6 times with consistent results. RFU, relative fluorescence units; RLU, relative luminescence units; AU, arbitrary units; UT, untreated.
