# Supplementary material for: Multimodal Regulation of NET Formation in Pregnancy: Progesterone Antagonizes the Pro-NETotic Effect of Estrogen and G-CSF
Source: Front Immunol. 2016 Dec 5;7:565. doi: 10.3389/fimmu.2016.00565 (PMC5136684; doi:10.3389/fimmu.2016.00565)
Supplement: Supplementary file 6 [file Figure_S4.PDF]

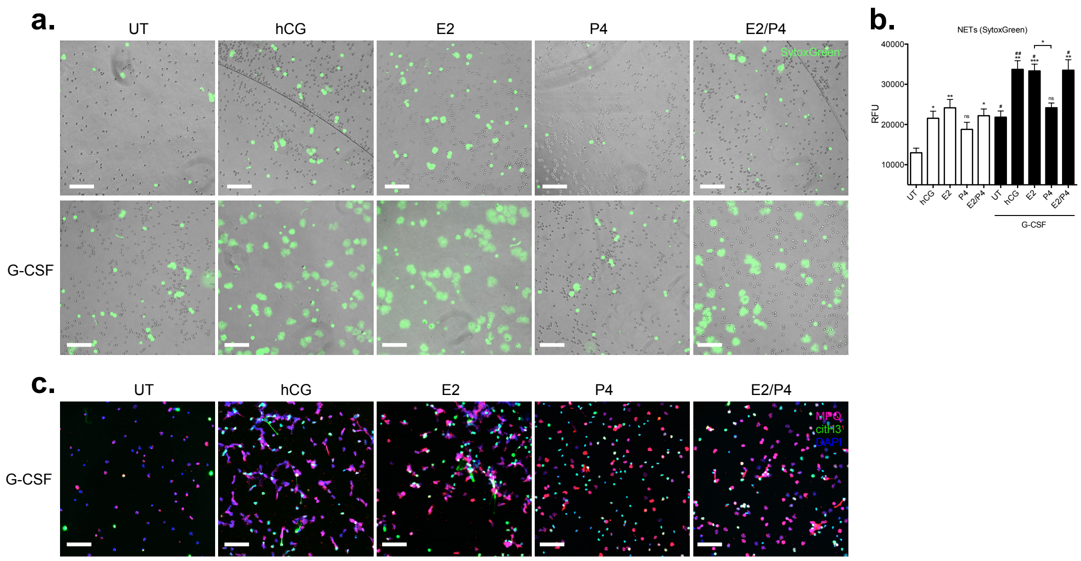

Fig. S4. Sex hormones differentially modulate G-CSF-driven neutrophil pro-NETotic priming. (**a**) *In vitro* NET formation assessed microscopically over a 3 hour timecourse with SytoxGreen DNA binding dye after without (upper panel) and with a 1 hour pretreatment with 12.5 ng/ml rhG-CSF (lower panel) and stimulation with the gestational hormones hCG (50 IU/ml), E2 (20 ng/ml), P4 (50 ng/ml) and the 1:1 combination of E2 and P4 for additional 2 hours. Magnification: 10x. Scale bars: 100 µm. (**b**) Fluorimetric quantification of extracellular DNA release of rhG-CSF-treated control neutrophils after addition of pregnancy hormones at concentrations given under (**a**). (**c**) Fluorescent immunostaining for MPO (red), citH3 (green) and DNA (blue) after a 3 hour *in vitro* co-culture of control neutrophils with rhG-CSF and addition of hormones at concentrations given under (**a**). Magnification: 20x; Scale bars: 50 µm. Data are presented as mean ± SEM. *P < 0.05, **P < 0.01, (one way ANOVA followed by Bonferroni's multiple comparison post-test). All experiments were performed at least 3 times with consistent results.
