# Supplementary material for: Multimodal Regulation of NET Formation in Pregnancy: Progesterone Antagonizes the Pro-NETotic Effect of Estrogen and G-CSF
Source: Front Immunol. 2016 Dec 5;7:565. doi: 10.3389/fimmu.2016.00565 (PMC5136684; doi:10.3389/fimmu.2016.00565)
Supplement: Supplementary file 7 [file Figure_S5.PDF]

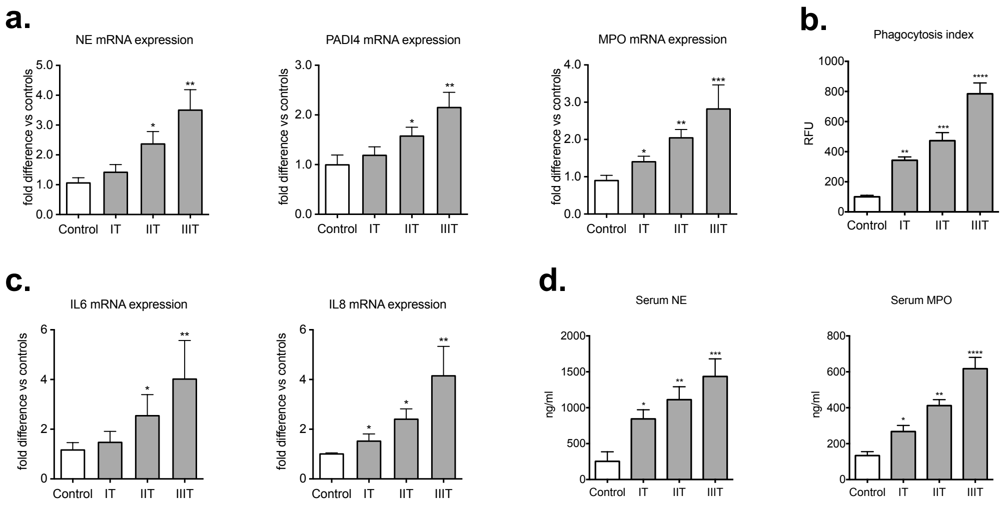

Fig S5. Signaling molecules involved in NET formation and neutrophil pro-NETotic priming are upregulated during pregnancy. (**a**) NE, PAD4 and MPO gene expression analysis by Taqman qRT-PCR in RNA samples obtained from healthy female controls and donors during pregnancy. (**b**) Phagocytic activity of neutrophils obtained from healthy female controls and donors during pregnancy. (**c**) IL6 and IL8 gene expression analysis by Taqman qRT-PCR in RNA samples obtained from healthy female controls and donors during pregnancy. (**d**) Detection of serum NE and MPO levels by ELISA. Data are presented as mean ± SEM. *P < 0.05, **P < 0.01, ***P < 0.001, ****P < 0.0001 (one or two way ANOVA followed by Bonferroni's multiple comparison post-test). All experiments were performed at least 6 times with consistent results. RFU, relative fluorescence units; IT, first trimester; IIT, second trimester; IIIT, third trimester.
