# Supplementary figures and images for: Multimodal Regulation of NET Formation in Pregnancy: Progesterone Antagonizes the Pro-NETotic Effect of Estrogen and G-CSF
Source: Front Immunol. 2016 Dec 5;7:565. doi: 10.3389/fimmu.2016.00565 (PMC5136684; doi:10.3389/fimmu.2016.00565)

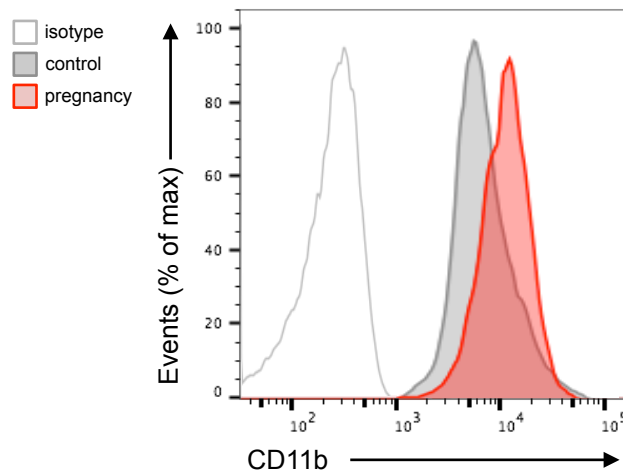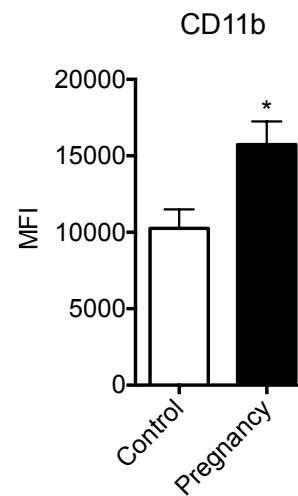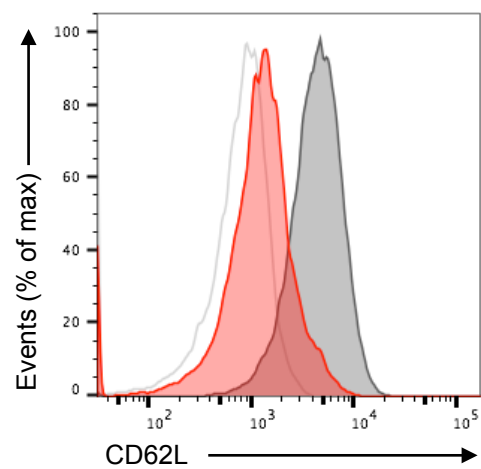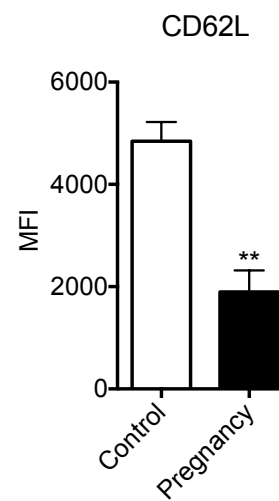

Supplement: Supplementary file 8 [file Figure_S6.PDF]
